# The progesterone to estradiol ratio predicts fear extinction in mice and humans

**DOI:** 10.1016/j.ynstr.2026.100823

**Published:** 2026-05-22

**Authors:** Jaime F. Nabás, Eric R. Velasco, David Fabregat-Safont, Élida Alechaga, Alex Gomez-Gomez, Marta Torrent, Mariana G. Fronza, Victoria Mueller, Mohammed R. Milad, Rafael Torrubia, Miquel A. Fullana, Katharina Schultebraucks, Oscar Pozo, Raul Andero

**Affiliations:** a Institut de Neurociències, Universitat Autònoma de Barcelona, Cerdanyola del Vallès, Barcelona, Spain; b Applied Metabolomics Research Group, Hospital del Mar Research Institute, Barcelona, Spain; c Environmental and Public Health Analytical Chemistry, Research Institute for Pesticides and Water (IUPA), Universitat Jaume I, Castelló, Spain; d Department of Psychiatry, NYU Grossman School of Medicine, New York, NY, USA; e Faillace Department of Psychiatry and Behavioral Sciences, McGovern Medical School, University of Texas Health Science Center at Houston, TX, USA; f Departament de Psiquiatria i Medicina Legal, Universitat Autònoma de Barcelona, Cerdanyola del Vallès, Barcelona, Spain; g Adult Psychiatry and Psychology Department, Institute of Neurosciences, Hospital Clinic, Barcelona, Spain; h Imaging of Mood- and Anxiety-Related Disorders Group, Institut d’Investigacions Biomèdiques August Pi i Sunyer (IDIBAPS), CIBERSAM, Barcelona, Spain; i Division of Healthcare Delivery Science, Department of Population Health, NYU Grossman School of Medicine, New York, NY, USA; j Centro de Investigación Biomédica En Red en Salud Mental (CIBERSAM), Instituto de Salud Carlos III, Madrid, Spain; k Unitat de Neurociència Traslacional, Parc Taulí Hospital Universitari, Institut d’Investigació i Innovació Parc Taulí (I3PT), Institut de Neurociències, Universitat Autònoma de Barcelona, Cerdanyola del Vallès, Spain; l ICREA, Barcelona, Spain

## Abstract

Sex differences in fear memory are well documented, yet the role of menstrual cycle phases and ovarian hormone dynamics remains unclear. Here, we investigated the individual and combined effects of progesterone and estradiol on fear acquisition and within-session fear extinction in both humans and mice. Human participants included men, women using oral contraceptives, and naturally cycling women across three menstrual phases. Our animal experiment included males and naturally cycling females tested across all estrous stages. Results in humans show an association between high estradiol levels and enhanced within-session extinction, whereas progesterone shows no direct effect. To further explore the relationship between sex hormones and within-session fear extinction, a machine-learning approach (Histogram-based Gradient Boosting Regression Tree) was followed. This showed that the facilitating effect of estradiol on within-session fear extinction is potentiated by its interactions with progesterone. Specifically, a high progesterone to estradiol ratio measured before extinction predicted enhanced extinction in both humans and mice. These findings identify the progesterone to estradiol ratio as a potential translational biomarker of fear extinction, with relevance to sex-informed treatments for fear-related disorders, and uncover a key memory process shared across species.

## 1 Introduction

Among fear-based disorders, we find post-traumatic stress disorder (PTSD), panic disorder, and specific phobias, which, at a clinical level, are characterized by altered fear learning (Flores et al., 2018). The impact of fear-based disorders is sex-dependent, with women showing double the lifetime prevalence of PTSD as men (Olff, 2017). To explain these differences, previous studies have investigated sex differences in fear learning, often putting a special focus on the modulatory role of sex hormones and their fluctuation throughout the menstrual cycle (Milad et al., 2009; Zeidan et al., 2011).

Despite most of the literature focusing on fear extinction (FE) recall, a few studies also focus on the effect of estradiol on within-session FE. Studies report reduced CS discrimination in FE training in healthy participants administered with estradiol, while others only see this effect in high estradiol PTSD patients (Glover et al., 2012; Kaczmarczyk et al., 2024). Regarding progesterone, its role has not been as thoroughly examined. Some studies have reported no significant effects of progesterone on fear acquisition (FA), within-session FE, or FE recall (Graham and Milad, 2013; Kaczmarczyk et al., 2024; Milad et al., 2010; Zeidan et al., 2011). However, other studies have reported differences in FE recall depending on trauma exposure, showing an impairment in FE recall in mid-luteal women, only when suffering from PTSD (Pineles et al., 2016).

Due to the wide use of oral contraceptive agents (OCA), it is of high importance to also consider their effects on emotional learning (United Nations Department of Economic and Social Affairs, 2022). Combined ethinilestradiol and progestin oral contraceptives act by inhibiting the production and secretion of the follicle-stimulating hormone and luteinizing hormone, impacting on estradiol and progesterone production (Rivera et al., 1999). When focusing on the effect of OCA on fear learning, some studies report impaired FE recall in OCA users (Graham and Milad, 2013; White and Graham, 2016). In contrast, others report reduced CS discrimination during FE training in OCA users, or even enhanced FA and within-session FE in PTSD women taking OCA (Bartholomew et al., 2022; Lonsdorf et al., 2015)

Despite the growing literature examining sex differences in human fear conditioning, there is still no agreement on many methodological aspects. There is no consensus about how the menstrual cycle should be monitored, and no general guidelines for the allocation of subjects in menstrual cycle phases (Stockhorst and Antov, 2016; Lonsdorf et al., 2015). Furthermore, when studies focus on hormonal levels in saliva, they tend to rely on ELISA kits, and due to the low levels present in this medium, these assays present important limitations in quantifying this steroid (Arslan et al., 2023). Moreover, human studies often perform FA and FE in the same session, which limits memory consolidation and focuses predominantly on short-term memory processes (Lonsdorf et al., 2017). Methodological differences are also seen in learning outputs. Skin conductance response (SCR) is the most common measure in human fear conditioning studies, which allows for comparison of the results with most of the literature (Merz et al., 2016; Milad et al., 2010; Pohlack et al., 2015; Seligowski et al., 2019). Meanwhile, fear-potentiated startle (FPS) is highly valuable due to its translational value, as well as sensitivity to individual differences (Andero et al., 2013; Chudoba and Dabrowska, 2025; Jovanovic et al., 2020; Lonsdorf et al., 2017). However, few studies have successfully combined both outcome measures. Given these gaps in the existing literature and methodological inconsistencies, we designed a FA and delayed FE protocol to test whether the menstrual cycle phase or sex steroid levels could modulate FA and FE in humans.

Individuals were classified by sex, oral contraceptive use, and menstrual cycle phase based on the timing of their last three menstruations. Participants were grouped into men, early follicular (EF) women, late follicular (LF) women, mid-luteal women (ML), and women using oral contraceptives. To measure sex steroid levels, saliva samples were collected and analyzed using a novel, highly sensitive liquid chromatography coupled with tandem mass spectrometry (LC-MS/MS) (Fabregat-Safont et al., 2024). Fear learning was assessed through subjective risk ratings (RR), SCR, and FPS. Our human experiment was replicated in mice, where freezing behavior was used as a fear learning index. Mice were divided by sex and estrous cycle phase, grouping them into five groups: males, proestrus females, estrus females, metestrus females, and diestrus females. To measure sex-steroid levels, blood samples were collected and analyzed via LC-MS/MS. We hypothesized that estradiol would enhance FE training, while progesterone would have no significant effect. Additionally, we also hypothesized that women in the oral contraceptive group would show similar FA and FE patterns to women in the LF group, due to high levels of exogenous and endogenous estrogens, respectively.

## 2 Materials and methods

### 2.1 Human participants

The study was approved by the Ethics Committee on Animal and Human Research from the Universitat Autònoma de Barcelona and conducted following the Declaration of Helsinki (2008) (UAB-CERec155). Participants were aged 18- 45, women had regular menstrual cycles, were not diagnosed with a mental, neurologic, or endocrinologic disorder, and did not take any psychotropic drug during at least one month before the study. Participants were classified as having a low trauma load/no trauma based on the self-reported lack of trauma- and stressor-related disorders. During an intake session (Day 0), those participants fulfilling inclusion criteria and accepting to participate signed informed consent forms, completed the Trait section of the Spanish Version of the State-Trait Anxiety Inventory (STAI-T) (Spielberger et al., 1982), and the Visual Analog Mood Scales (VAMS) (Stern et al., 1997), and proceeded to saliva sample collection, and in the case of women, vaginal cytology auto collection. Participants were asked to abstain from alcohol for 24h, caffeine for 12h, and food for 2h before each experimental session. Thirteen participants declined to participate in the day-2 session, and two participants were excluded due to technical problems. Two participants were excluded from the analyses due to the use of non-oral contraceptive methods. The final sample consisted of 128 participants (95 women, 33 men; mean age = 23.4, SD = 4.6). Participants completing the study received 20€ as compensation.

### 2.2 Study overview

All participants underwent a two-day experimental protocol where SCR, FPS, and subjective RR were assessed during FA (day 1) and FE training (day 2, 24h after). Saliva samples and vaginal cytologies were collected in both sessions.

### 2.3 Stimuli and procedure

The CSs were two geometric figures (a square and a circle) presented on a computer screen for 8000 ms. The unconditioned stimulus (US) was an electric shock of 100 ms delivered to the non-dominant forearm. The US was generated by a stimulator (Grass Instruments S48, USA), isolated (SIU5), and transmitted via a constant current unit (CCU1) to a bipolar bar electrode (EP10-621, Technomed Europe, Netherlands). The startle probe was a 50 ms burst of white noise at 102 dB, with near-instantaneous rise time, delivered binaurally through high-fidelity headphones. RR for each CS were obtained using a scale presented on the screen (0 = no risk, 9 = high risk) during each CS presentation. Stimulus presentation and timing were controlled with the software Affect 4.0 (KU Leuven, Belgium) (Spruyt et al., 2009).

In experimental day 1 FA, participants gave a 2-ml saliva sample (before FA) and a vaginal cytology (only women, after FA), completed the state section of the STAI (STAI-S), the recording and shock electrodes were attached, the intensity of the US was adjusted (to an intensity that was “highly uncomfortable but not painful”), and the headphones for the startle probes were placed. Participants were asked to assess the risk of shock for each CS presentation using a 0-9 scale. During the *fear pre-acquisition*, two non-reinforced presentations of each CS occurred, followed by a habituation phase with 6 presentations of the startle probe alone (NA). During FA, one of the CS was paired to the US 500ms before its offset (CS+) at an 83% reinforcement rate, while the other CS was not (CS-). A fixation cross appeared during intertrial intervals (ITIs). Trial order was pseudorandomized and counterbalanced across CS, with no more than two presentations of a specific CS occurring in a row. Startle probes appeared 1000 ms before the end of the CS presentation in 66% of the acquisition trials. ITIs and inter-probe intervals ranged from 10 to 14 s, and 18-22 s, respectively. The acquisition phase consisted of 12 presentations of each of the CS+, CS-, and NA. The figure used as CS+ was counterbalanced between subjects. After the task, participants completed the STAI-S, rated the discomfort associated with the startle probe and the US, and answered a question to assess contingency awareness (Florido et al., 2024; Velasco et al., 2025).

On day 2 (FE), participants followed the same recording procedures as on day 1, excluding US calibration. They were instructed to recall what they experienced in the previous session. Samples were collected following the same protocol used on day 1 (the saliva sample was obtained before FE, and the vaginal cytology after FE, only from women). The experimental procedure was identical to day 1, with the differences that no pre-acquisition block or USs were administered during day 2 (For a schematic representation of the task's structure, see Supplementary Fig. 1). For physiological recordings and response definition, see supplementary materials and methods.

### 2.4 Menstrual cycle phase allocation

The last three menstrual period dates were collected for each woman, along with self-reported cycle regularity/length. Menstrual cycle phase length was calculated following Bull et al., 2019. The EF phase was considered from the first day of menstruation to −6 days before the approximate ovulatory date. The LF was considered from −5 to +1 days of the approximate ovulatory day. The ML was considered from the +2 day after the approximate ovulatory date −3 days to the end of the cycle. Only two women fell into the late luteal window (the last 2 days of the cycle) and were included in the mid-luteal group. These three phases were selected as experimental groups due to their different hormonal profiles.

### 2.5 Vaginal cytology processing

Three vaginal cytologies were self-performed by each woman (one per session). Women were asked to insert a sterile cotton swab 5 cm, roll it on the vaginal walls for 10 s, and then return it to the researcher. The samples were immediately transferred to adhesion slides and sprayed with an ethanol-based fixative solution (M-FIX, Germany). For staining, an adapted version of the Papanicolau stain (Papanicolaou, 1933) was used, which includes Hematoxilin, Eosin, and Orange G. Slides of free-cycling women were analyzed under an optical microscope Eclipse 80i (Nikon, Japan). Pictures were scored and classified blindly to the experimental group. For sample classification, see supplementary materials and methods.

### 2.6 Liquid chromatography – mass spectrometry hormonal quantification

Two different LC-MS/MS protocols were used. Both methods were validated based on the European Medicines Agency (EMA), demonstrating their suitability for the determination of salivary hormones. Estradiol was quantified following a novel method for salivary estradiol determination (Fabregat-Safont et al., 2024), whereas progesterone, testosterone, cortisol, and other steroid hormones were quantified following Gomez-Gomez et al. (2020). For detailed LC-MS/MS protocols, see supplementary materials and methods.

### 2.7 Mice

Ethics protocols approved for the experiments in mice ref. CEEAH 3995, 6205, 6228, 6229, 6230, 6231, 6223, and 6235. All procedures were approved by the Committee of Ethics of the Universitat Autònoma de Barcelona and the Generalitat de Catalunya. They were also carried out as per the European Communities Council Directive (2010-63-UE) and Spanish legislation (RD 53/2013). Male and naturally cycling female C57BL/6 mice purchased from Charles River (Barcelona, Spain), or bred in our colony, were used. Mice bought from Charles River were kept undisturbed in the housing facility for 7 days before any handling or experimental procedures were performed. Mice were aged 9 – 17 weeks and lived group-housed (two to six mice per cage) in the vivarium, in a 12-h light/12-h dark cycle, with controlled temperature (22 ± 3 °C) and humidity (55 ± 10%), and ad libitum food and water. Male and female mice were housed separately in the same room. (For a schematic representation of the experimental paradigms’ structures, see Supplementary Fig. 1)

### 2.8 Estrous cycle monitoring

To assess the phase of the estrus cycle, vaginal cytology smears were performed daily for all female mice following Florido et al. (2021). The estrus cycle was monitored for an average of 10.42 days (SD = 7.56). All vaginal smear samples were collected between 8 and 11:00 a.m. through a vaginal lavage with 10 μl of standard NaCl 0.9% (w/v) solution. Once dry, slides were stained for 5 min in Cresyl Violet Acetate (C5042, Sigma-Aldrich, Spain) 0.1% (v/v), washed twice for 1 min in distilled water, and read under brightfield microscopy. For estrous cycle classification, see supplementary materials and methods.

### 2.9 Cued fear conditioning

To perform FA and FE, the tone and shock delivery were managed by the Freezing v1.3.04 software (Panlab-Harvard, Barcelona, Spain). The fear chamber used for FA and FE consisted of a methacrylate black box with a transparent front door (25 × 25 × 25 cm^3^) placed inside another sound-attenuating box with dimensions of 67 × 53 × 55 cm^3^. Two days before FA, mice were habituated to the fear chamber for 5 min each day. For FA, a footshock (1 s, 0.3 mA) served as the US, and a tone (30 s, 75 dB, 6 kHz) served as the conditioned stimulus (CS). The FA paradigm included a 5-min habituation period before the onset of the first tone. Mice then received 5 trials of the CS and US presented simultaneously with an intertrial interval (ITI) of 3 min, with 3 additional minutes after the final trial. The FE session was conducted 24 h after FA for 86.9% of the mice (48 h after for 7.1%, and 72 h for 6.1%). Mice were placed in the chamber for 5 min of habituation before being presented with 30 trials of the CS, with an ITI of 30 s and an additional 30 s following the last trial. Freezing behavior served as a fear measure. Each animal was recorded with a video camera positioned on the roof of the box. Freezing behavior was analyzed using the ezTrack behavior tracking code (Pennington et al., 2019), with a threshold of 75 and a minimum duration of 5 frames.

FA and FE were conducted under different environmental conditions to minimize contextual freezing during FE. For FA, a white light source and a grid floor were used, and the chamber was cleaned with ethanol (70% v/v) between sessions. For FE, a red light source and a grey floor (without bars) were used, and the chamber was cleaned with CR36-bronopol (0.26% v/v) between sessions. Additionally, transportation routes between the vivarium and testing room, and the researcher's glove material were changed between FA and FE. Behavioral testing was always conducted during the light phase of the cycle (FA 12:22h ± 1:16; FE 13:09h ± 1:46) (Florido et al., 2024; Velasco et al., 2022).

### 2.10 Tail blood collection

The collection procedure was performed according to Florido et al. (2021) before FE. Once the animal was immobilized, the lateral tail vein was located, and a small cut was made using a scalpel. The animal was then placed on top of a wire-bar rack to allow free movement, while a capillary tube was used to collect blood drops. Once the required volume was obtained, mice were left undisturbed for 2 h in their home cage. The sample volume was 150 - 200 μL. All mice were habituated to the procedure at least 3 days before FE by performing all steps except the incision.

### 2.11 Heart blood collection

Right after FE, mice were anesthetized with isoflurane. Once breaths were spaced by a second, animals were removed from the anesthesia chamber and their heads were placed inside a tube with cotton wool soaked in isoflurane to maintain anesthesia. Then, an incision on the peritoneal cavity was made, the diaphragm was cut, and the heart was exposed. For blood collection, a 25-gauge needle attached to a syringe coated with lithium heparin salt dissolved in water (40 mg/mL) was inserted into the left ventricle. After collection, samples were transferred to an anticoagulant-treated tube.

### 2.12 Blood sample processing

Tubes were centrifuged for 15 min at 8000 × g, 4 °C. Supernatant (plasma) was immediately transferred into a clean polypropylene tube and frozen at −80 °C until analysis. For detailed mouse LC-MS/MS protocols, see supplementary materials and methods.

### 2.13 Data analysis

Normalization was performed on both SCR (square root transformation) and FPS (T-scores) data. FA and FE training were analyzed separately for each session through repeated measures ANOVA, with stimulus and block as within-subjects factors, and group as between-subjects factor. Significant (p < 0.05) or near-significant (p ≤ 0.1) interactions were followed by pairwise comparisons. Mean values for age, STAI-T, US intensity, US discomfort, and startle discomfort were compared between groups using one-way ANOVA, and frequencies using Chi-squared tests. Both men and women were included in the analysis of hormone data. Spearman's correlations were performed between hormone levels and CS discrimination scores (CS+ – CS-). Progesterone-Estradiol (P/E2) ratios were calculated by dividing progesterone by estradiol levels when both were reported pg/mL, following (Seligowski et al., 2020). A Fisher's exact test was performed to study the differences between a cytology-based classification and a menstrual cycle length-based classification. These statistical analyses were performed with IBM SPSS 25.0.

To explore the predictive capacity of different variables over FE, we applied a Histogram-based Gradient Boosting Regression Tree (HGBRT). This analysis was performed in Python 3.9.21. The outcome variable was the CS discrimination scores during the FE training session. The predictor variables included demographic and clinical characteristics, previous traumatic experience, state and trait anxiety, hormone levels at the time of FE, and menstrual cycle phase, among others. The importance of each variable in predicting the outcome variable was determined using SHapley Additive exPlanation (SHAP) values.

In the animal experiment, we performed repeated measures ANOVA with CS (FA) or block (FE) as a within-subjects factor. Group was included as a between-subjects factor. Significant (p < 0.05) or near-significant (p ≤ 0.1) interactions were followed by pairwise comparisons. We report η2 as an estimate of effect size. For hormone data, the same approach as in humans was followed by including both male and female mice. First, Spearman's correlations between hormone levels and freezing during FE were performed. A HGBRT was used as a predictive model. In this case, the outcome variable was the percentage of the CSs spent freezing across the FE session. The predictor variables included sex, age, weight, number of mice housed in the same cage, estrous cycle phase, time of FA and FE, volume of plasma extracted before FE, and hormone levels, among others. For extended data analysis, see supplementary materials and methods.

### 2.14 AI tools

ChatGPT-4 was used for summarization and assistance in writing specific parts of the abstract, introduction, and discussion. The resulting text was always further edited by the researcher.

## 3 Human results

### 3.1 Participants’ characteristics

Of the 128 participants who completed the study, 33 were men (25.8%) and 95 were women (74.2%). Women were allocated into 4 groups based on the use of oral contraceptives or the phase of the menstrual cycle they were in on the experimental days. 24 participants used OCA (18.8%). Free-cycling women were classified as follows: 23 in the EF phase (18%), 30 in the LF phase (23.4%), and 18 in the ML phase (14.0%). There were no significant differences among groups for age, STAI-T, US intensity, US trials required for calibration, US discomfort, startle discomfort, and proportion of contingency-aware participants (Supplementary Table 1).

### 3.2 Whole sample fear acquisition and extinction training

We first verified the validity of the two-day paradigm across the entire sample by comparing the five groups on FPS, SCR, and RR by repeated measures ANOVAs with group as a between-subjects factor. These analyses showed successful FA and FE learning in both physiological measures, FPS and SCR, with no differences between groups. On RR, successful subjective FA was found with no group differences. However in FE training, a stimulus∗block∗group interaction (F(6.2,191.5) = 2.698, p = 0.014, ηp^2^ = 0.081) emerged, showing that the significant discrimination between CS+ and CS- in the last block of extinction is exclusively driven by Men (p < 0.001) (see Supplementary Fig. 2 for whole sample results, and Supplementary Figs. 3, 4, and 5 for group analysis in FPS, SCR, and RR, respectively).

### 3.3 Role of estradiol on fear acquisition and extinction

To isolate the effect of endogenous estradiol, we first compared women in the EF phase (low estradiol) with those in the LF phase (high estradiol) using repeated measures ANOVAs with group (EF/LF) as a between-subjects factor. Both groups successfully acquired fear during FA across all three measures. For FPS, there was a significant main effect of stimulus (F(2,98) = 36.168, p < 0.001, ηp^2^ = 0.425) with significant CS + potentiation (p < 0.001) and CS discrimination (p = 0.009), indicating successful fear learning. Similarly, SCR showed a significant main effect of stimulus (F(1,51) = 17.958, p < 0.001, ηp2 = 0.260) with clear CS discrimination (p < 0.001) (Supplementary Fig. 4b and c). In RR, a significant main effect of stimulus (F(1,51) = 332.182, p < 0.001, ηp2 = 0.867) was also found, again reflecting significant CS discrimination (p < 0.001) (Supplementary Fig. 5b and c). Across all measures, there were no significant interactions or main effects of group, indicating that EF/LF phases did not influence fear acquisition.

**Fig. 1:**
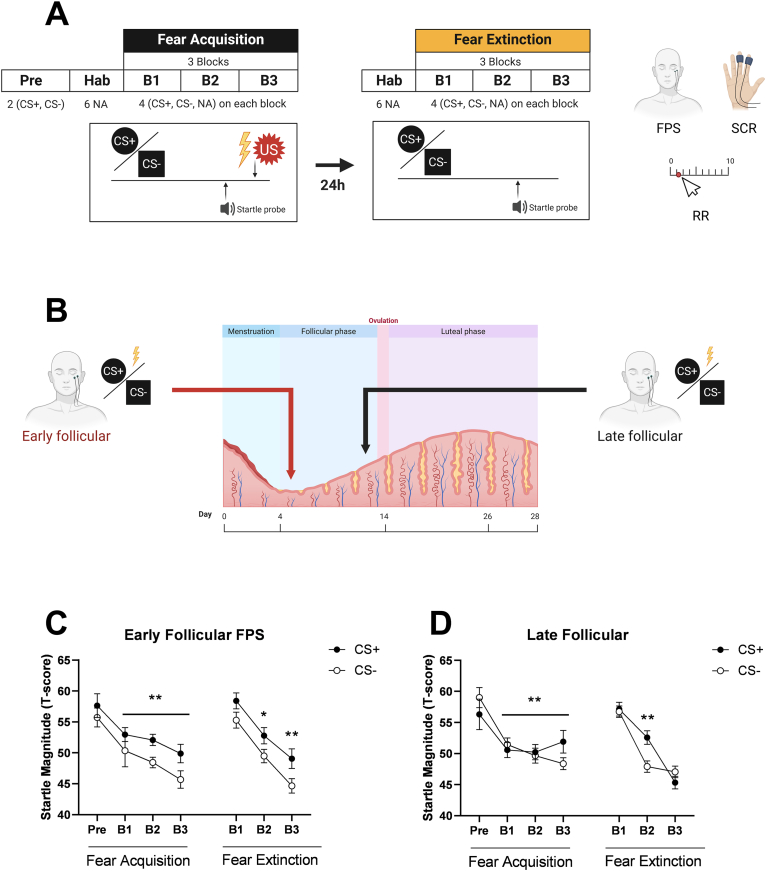
**Fear acquisition and extinction in human early follicular and late follicular groups. Fear-potentiated startle.** Panel A shows schematic representation of experimental design, Panel B shows menstrual cycle allocation of modeled groups, Panel C shows the early follicular group, and Panel D shows the late follicular group. B1, B2, B3: block, CS+: reinforced CS, CS-: non-reinforced CS, pre: pre-acquisition trials. ∗ Indicates CS discrimination (e.g., higher responses to CS+>CS-). ∗ = p < 0.05, ∗∗ = p < 0.01, ∗∗∗ = p < 0.001. Horizontal line above blocks indicates main effect stimulus.

A distinct pattern emerged during extinction. In FPS, a stimulus∗block interaction (F(4,204) = 6.796, p < 0.001, ηp^2^ = 0.118) showed that CS discrimination was only achieved in the second block of FE (B1, p = 0.161; B2, p < 0.001; B3, p = 0.190). Interestingly, a near-significant stimulus∗block∗group interaction (F(4,204) = 2.320, p = 0.058, ηp^2^ = 0.044) indicated that LF women fully extinguished their fear response by block 3 (p = 0.199), whereas EF women continued to discriminate between CS+ and CS- through the end of the session (p = 0.005) (Fig. 1c and d). In SCR, a main effect of stimulus was observed (F(1,50) = 22.671, p < 0.001, ηp^2^ = 0.312) with significant CS discrimination (p < 0.001) (no significant interactions or main effect of group) (Supplementary Fig. 4b and c). In RR, a main effect of stimulus (F(1,51) = 71.009, p < 0.001, ηp^2^ = 0.582) with significant CS discrimination (p < 0.001) was observed, as well as a stimulus∗block interaction (F(1.5,76.4) = 100.100, p < 0.001, ηp^2^ = 0.662), which showed that CS discrimination disappeared by block 3, reflecting full subjective extinction (B1, p < 0.001; B2, p < 0.001; B3, p = 0.061) (no significant interactions or main effect of group) (Supplementary Fig. 5b and c).

Because the menstrual phase is an indirect measure of hormonal status, we separately investigated the direct influence of estradiol through the LC-MS-quantified levels of estradiol in saliva. For hormone levels across groups, see Table 1. For estradiol levels across the menstrual cycle, see Supplementary Fig. 6. For progesterone levels across the menstrual cycle, see Supplementary Fig. 7. Spearman correlations between estradiol and CS discrimination were not significant for FA or FE, either at the whole-session level or block by block (Supplementary Figs. 8 and 9). Therefore, based on previous literature, a median split approach was used to divide participants into high and low hormone groups, and those groups were then compared using repeated-measures ANOVAs (Graham et al., 2017; Milad et al., 2010; Zeidan et al., 2011).

**Table 1:** Human hormone levels.

	Men (33)	EF (23)	LF (30)	ML (18)	OCA (24)	*p-value*
Estradiol						
Day 0	0.0004 ± 0.16	0.0004 ± 0.30	0.0004 ± 0.15	0. 0007 ± 0.32∗∗∗	0.0003 ± 0.20	<0.001
Day 1	0.0004 ± 0.22∗∗∗	0.0006 ± 1.14∗∗	0.0012 ± 0.76	0.0014 ± 1.26	0.0003 ± 0.14∗∗∗	<0.001
Day 2	0.0004 ± 0.22∗∗∗	0.0006 ± 0.85∗∗∗	0.0014 ± 1.13	0.0010 ± 0.38#	0.0003 ± 0.17∗∗∗	<0.001
Progesterone						
Day 0	0.009 ± 0.028	0.006 ± 0.013	0.002 ± 0.002	0.017 ± 0.033	0.002 ± 0.002	0.095
Day 1	0.005 ± 0.01°°°	0.004 ± 0.004°°°	0.005 ± 0.006°°°	0.06 ± 0.06	0.003 ± 0.007°°°	<0.001
Day 2	0.005 ± 0.01°°°	0.003 ± 0.003°°°	0.009 ± 0.01°°°	0.07 ± 0.06	0.002 ± 0.002°°°	<0.001
Testosterone						
Day 0	7.61 ± 4.24	0.47 ± 0.24ˆˆˆ	0.52 ± 0.34ˆˆˆ	0.63 ± 0.28ˆˆˆ	0.39 ± 0.13ˆˆˆ	<0.001
Day 1	7.84 ± 5.29	0.66 ± 0.69ˆˆˆ	0.84 ± 0.64ˆˆˆ	0.89 ± 0.79ˆˆˆ	0.29 ± 0.16ˆˆˆ	<0.001
Day 2	7.41 ± 4.84	0.49 ± 0.22ˆˆˆ	1.06 ± 1.78ˆˆˆ	0.85 ± 0.82ˆˆˆ	0.30 ± 0.20ˆˆˆ	<0.001

Data is presented as mean ± SD. For estradiol ∗ indicates significant differences between the marked group and LF (reference group with highest estradiol) ∗ = p < 0.05, ∗∗ = p < 0.01, ∗∗∗ = p < 0.001. Sample sizes are specified in parentheses next to the group names. For progesterone ° indicates significant differences between the marked group and mid-luteal (reference group with highest progesterone) ° = p < 0.05, °° = p < 0.01, °°° = p < 0.001. For testosterone ˆ indicates significant differences between the marked group and males (reference group with highest testosterone) ˆ = p < 0.05, ˆˆ = p < 0.01, ˆˆˆ = p < 0.001. # indicates trend level significance (p = 0.05). Hormone levels are reported in ng/ml. EF = early follicular. LF = later follicular. ML = mid-luteal. OCA = oral contraceptive users. Hormone levels are reported in ng/ml.

**Fig. 2:**
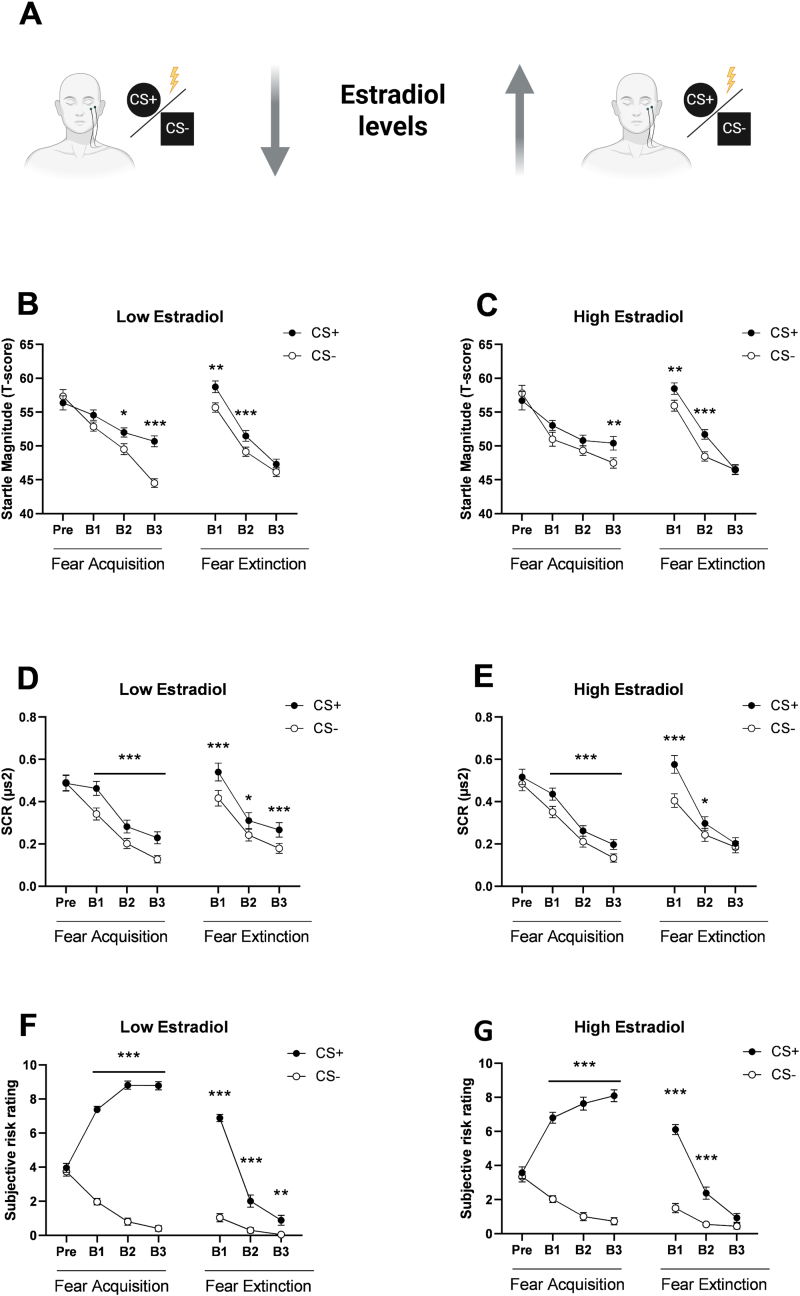
Fear acquisition and extinction in human low-estradiol and high-estradiol groups. Fear-potentiated startle, skin conductance response, and subjective risk ratings. Panel A shows schematic representation of the analysis conducted, Panel B shows FPS for the low-estradiol group, Panel C shows FPS for the high-estradiol group, Panel D shows SCR for the low-estradiol group, and Panel E shows SCR for the high-estradiol group, Panel F shows RR for the low-estradiol group, and Panel G shows RR for the high-estradiol group. B1, B2, B3: block, CS+: reinforced CS, CS-: non-reinforced CS, pre: pre-acquisition trials. ∗ Indicates CS discrimination (e.g., higher responses to CS+>CS-). ∗ = p < 0.05, ∗∗ = p < 0.01, ∗∗∗ = p < 0.001. Horizontal line above blocks indicates main effect stimulus.

With the median-split classification, high/low estradiol showed successful FA, similarly between groups, though some subtle timing differences emerged. FPS showed a main effect of stimulus (F(2,244) = 129.400, p < 0.001, ηp^2^ = 0.515) with CS + potentiation and CS discrimination (both p < 0.001), indicating successful FA. A significant stimulus∗block∗group interaction (F(4,488) = 2.411, p = 0.048, ηp^2^ = 0.019) showed that, despite both groups reaching significant CS discrimination by the end of the session, the low estradiol group showed this significant discrimination earlier, by block 2 (Low estradiol: B1, p = 0.154; B2, p = 0.022; B3, p < 0.001; High estradiol: B1, p = 0.080; B2, p = 0.157; B3, p = 0.008) (Fig. 2b and c). SCR measures were equivalent between groups, both denoting FA (main effect of stimulus: F(1,123) = 71.410, p < 0.001, ηp^2^ = 0.367) and significant CS + potentiation and CS discrimination (both p < 0.001, no significant interactions or main effect of group) (Fig. 2d and e). RR showed a main effect of stimulus (F(1,115) = 788.665, p < 0.001, ηp^2^ = 0.873) with CS + potentiation and CS discrimination (both p < 0.001), also denoting FA. A significant stimulus∗group interaction (F(1,115) = 4.378, p < 0.039, ηp2 = 0.037) was found, indicating that despite slight differences in the magnitude of discrimination between groups, both groups clearly differentiated CS+ from CS- (p < 0.001) (Fig. 2f and g).

FE, in contrast, revealed a consistent facilitating role of estradiol. In FPS, a main effect of stimulus (F(2,248) = 118.205, p < 0.001, ηp^2^ = 0.488) and a stimulus∗block interaction (F(4,496) = 17.071, p < 0.001, ηp^2^ = 0.121) showed that CS discrimination disappeared by block 3 (B1, p = 0.002; B2, p < 0.001; B3, p = 0.389), without group differences (Fig. 2b and c). However, in SCR a significant main effect of stimulus (F(1,123) = 46.002, p < 0.001, ηp^2^ = 0.272) combined with a stimulus∗block∗group interaction (F(2,246) = 3.048, p = 0.049, ηp^2^ = 0.024) emerged, demonstrating that the low estradiol group had significant CS discrimination by the end of FE training (p < 0.001), while the high estradiol group had fully extinguished (Low estradiol: B1, p < 0.001; B2, p = 0.011; B3, p < 0.001; High estradiol: B1, p < 0.000; B2, p = 0.040; B3, p = 0.447) (Fig. 2d and e). RR reinforced this result. Significant main effect of stimulus indicated successful FE learning (F(1,115) = 116.244, p < 0.001, ηp^2^ = 0.591), however, a significant stimulus∗block∗group interaction (F(1.6,180.7) = 3.687, p < 0.037, ηp^2^ = 0.031) showed that by the end of the session, only high estradiol participants rated the two CS equally (Low estradiol: B1, p < 0.001; B2, p < 0.001; B3, p = 0.003; High estradiol: B1, p < 0.001; B2, p < 0.001; B3, p = 0.084) (Fig. 2f and g).

Altogether, these findings reveal a consistent facilitating role of estradiol on within-session FE that is not detectable during acquisition. FA was equivalent regardless of menstrual phase or estradiol level, with only minor differences in the timing of CS discrimination. During FE, however, both the indirect (EF vs. LF phase) and the direct (high vs. low estradiol) classifications converged on the same pattern: women with higher estradiol extinguished their conditioned fear responses faster and more completely than those with lower estradiol, with particularly robust findings in SCR and RR, but also discernible as a trend in FPS.

### 3.4 Role of progesterone in fear acquisition and extinction

To explore the role of progesterone on fear learning, the same approach as for estradiol was followed (for progesterone levels across the menstrual cycle, see Supplementary Fig. 7). No significant correlations between progesterone and CS discrimination were found in FA or FE (Supplementary Figs. 8 and 9).

When participants were split into high- and low-progesterone groups, FA was equivalent across the three measures and showed no group effects. FPS revealed a main effect of stimulus (F(2,248) = 132.580, p < 0.001, ηp^2^ = 0.517) with CS discrimination and CS + potentiation (both p < 0.001) (Supplementary Fig. 10a and b). SCR showed a main effect of stimulus (F(1,125) = 68.502, p < 0.001, ηp^2^ = 0.354) with significant CS discrimination (p < 0.001) (Supplementary Fig. 11a and b). Similarly, RR showed a main effect of stimulus (F(1,126) = 854.982, p < 0.001, ηp^2^ = 0.872) with significant CS discrimination (p < 0.001) (Supplementary Fig. 12a and b). These results indicate successful FA in the three analyzed measures (FPS, SCR, and RR) with no significant interactions or main effect of group.

FE was also comparable between low/high progesterone groups. In FPS, a main effect of stimulus (F(2,252) = 113.985, p < 0.001, ηp^2^ = 0.475) together with a stimulus∗ block interaction (F(4,504) = 17.665, p < 0.001, ηp^2^ = 0.123) reflected successful extinction, with CS discrimination disappearing by block 3 (B1, p = 0.002; B2, p < 0.001; B3, p = 0.387) and no interaction or main effect of group (Supplementary Fig. 10a and b). SCR showed a main effect of stimulus (F(1,125) = 45.440, p < 0.001, ηp^2^ = 0.267) without significant interaction or main effect of group (Supplementary Fig. 11a and b). RR also showed a main effect of stimulus (F(1,126) = 190.675, p < 0.001, ηp^2^ = 0.602), accompanied by a group∗block interaction (F(2,252) = 515.458, p < 0.001, ηp^2^ = 0.804). Pairwise comparisons revealed that both groups reduced CS discrimination comparably across all blocks (p < 0.001) (Supplementary Fig. 12a and b). Therefore, in isolation, progesterone levels showed no detectable influence on FA or within-session FE.

### 3.5 Combined effect of estradiol and progesterone on fear acquisition and extinction

**Fig. 3:**
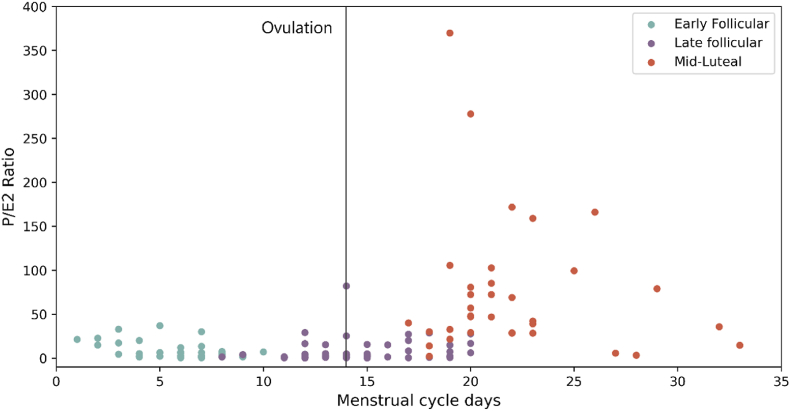
**Human progesterone/estradiol ratios throughout the menstrual cycle.** The Y axis represents progesterone/estradiol ratios, and the X axis represents the days of the menstrual cycle in which the saliva samples were collected. Blue dots represent participants in the early follicular phase, purple dots represent subjects in the late follicular phase, and red dots represent participants in the mid-luteal phase.

Because estradiol and progesterone fluctuate in partially coordinated ways across the cycle, prior work has proposed the P/E2 ratio as a more integrative biomarker of hormonal state relevant to fear processing (Seligowski et al., 2020) (See Fig. 3 for P/E2 ratio distribution across the menstrual cycle). The same approach used for individual hormones was followed, and no significant correlations between P/E2 ratios and CS discrimination in FA or FE were found (Supplementary Figs. 8 and 9).

During FA, FPS showed a main effect of stimulus (F(2,244) = 128.232, p < 0.001, ηp^2^ = 0.512) with significant CS discrimination and CS + potentiation (both p < 0.001), denoting fear learning (Supplementary Fig. 10c and d). SCR showed a main effect of stimulus (F(1,123) = 68.619, p < 0.001, ηp^2^ = 0.358) with CS discrimination (p < 0.001) (Supplementary Fig. 11c and d). RR confirmed successful FA with a main effect of stimulus (F(1,124) = 869.471, p < 0.001, ηp^2^ = 0.875) with CS discrimination (p < 0.001) (Supplementary Fig. 12c and d). Therefore, successful FA were observed in FPS, SCR and RR with no significant interactions or main effects of the group, suggesting that the P/E2 ratio does not influence fear learning.

The absence of group effects persisted during fear extinction (FE) across FPS, SCR, and RR measures. In FPS, a main effect of stimulus (F(2,248) = 118.985, p < 0.001, ηp^2^ = 0.490) and a stimulus∗block interaction (F(4,496) = 17.153, p < 0.001, ηp^2^ = 0.122) indicated that CS discrimination disappeared by block 3 (B1, p = 0.002; B2, p < 0.001; B3, p = 0.405) (Supplementary Fig. 10c and d). SCR showed a main effect of stimulus (F(1,123) = 45.811, p < 0.001, ηp^2^ = 0.271) (Supplementary Fig. 11c and d), and RR mirrored this pattern (F(1,124) = 185.929, p < 0.001, ηp^2^ = 0.600) (Supplementary Fig. 12c and d). No significant interactions or main effect of group were found in the three measures. These results indicate that conventional analyses based on high/low splits of the P/E2 ratio did not reveal a linear relationship with extinction learning, suggesting that any influence of the progesterone/estradiol balance is non-linear or interacts with other variables not captured by dichotomisation.

### 3.6 Predictive machine learning model

Given the ability of machine-learning approaches to detect non-linear patterns and interactions that can be obscured by conventional ANOVA, we performed a HGBRT analysis to assess hormones’ predictive capacity over FE training.

**Fig. 4:**
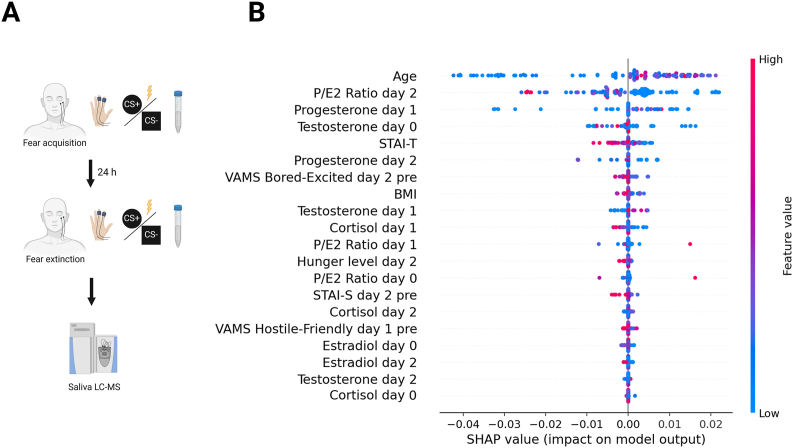
**SHAP summary plot showing the top 20 features with the most influence on the model prediction of fear extinction in both men and women, measured using skin conductance response.** Panel A shows a schematic representation of the experimental design, Panel B shows the HGBRT outcome. The higher the SHAP, the higher the probability of higher CS discrimination SCR values. Features are first sorted by their global impact (y-axis). For each individual in the sample, a dot represents the attribution value for each feature from low (blue) to high (red).

**Fig. 5:**
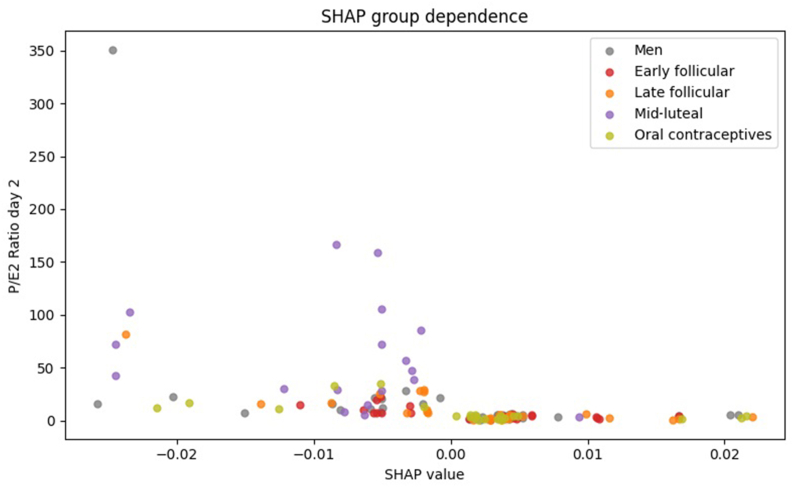
**SHAP dependence plot in humans. Interaction of P/E2 ratio with the different menstrual cycle phases.** The distribution of SHAP values across the different phases shows the importance of the mid-luteal group for the relationship between the P/E2 ratio and fear extinction, as this group is the most concentrated on the negative values.

The trained model achieved a Mean Absolute Error (MAE) 0.1042 (SD = 0.0127), a Root Mean Square Error (RMSE) of 0.1375 (SD = 0.0204), and a mean R^2^ of 0.0151 (SD = 0.069). Fig. 4 represents which variables have the strongest influence on the model's prediction. Critically, SHAP values revealed that hormonal variables, specifically the P/E2 ratio, were among the top predictors of CS discrimination in SCR during FE, alongside demographic and psychological features (Fig. 4). We conducted additional statistical analyses to explore group-specific effects. An Ordinary Least Squares (OLS) regression with SHAP values as the dependent variable, and ML group and P/E2 ratios as predictors showed a significant effect of the ML group (p = 0.015) and a marginal trend toward significance of the ML∗P/E2 ratio interaction (p = 0.100). This suggests that the relationship between P/E2 and SHAP values might differ slightly, but not significantly, based on ML group belonging (Fig. 5). This proves the predictive profile of our variables to be a sample-wide characteristic, not limited to a specific group. These variables showed no significant predictive ability for FPS data (Supplementary Fig. 13).

## 4 Supplementary testosterone and cortisol analyses

Additionally, the same statistical approaches were applied for testosterone and cortisol data. Testosterone levels were positively correlated with CS discrimination in SCR during the third block of FA (Supplementary Fig. 9). Nevertheless, no significant group effects were found when modeling testosterone or cortisol group in repeated measures ANOVA across any of the three outcomes: FPS, SCR and RR (Supplementary Figs. 10, 11, and 12, e-h).

### 4.1 Role of oral contraceptives in fear acquisition and extinction

Because combined oral contraceptives produce high circulating levels of synthetic estrogens that may functionally mimic the endogenous estradiol peak of the LF phase, we directly compared LF and OCA women. Both groups successfully acquired fear in all three measures. In FPS, a main effect of stimulus (F(2,102) = 47.616, p < 0.001, ηp^2^ = 0.483) with CS + potentiation (p < 0.001) and CS discrimination (p = 0.006), and a stimulus∗block∗group interaction (F(4,204) = 3.078, p = 0.017, ηp^2^ = 0.057) showed that both OCA women (B1, p = 0.124; B2, p = 0.865; B3, p < 0.001) and LF women (B1, p = 0.587; B2, p = 0.737; B3, p = 0.040) reached significant discrimination only in block 3. In block 3, discrimination was higher in the OCA group (7.77) than in the LF group (3.53), driven by a drop in CS- responsivity in OCA women (Supplementary Fig. 14a and b). SCR and RR confirmed successful FA with a main effect of stimulus (F(1,52) = 29.075, p < 0.001, ηp^2^ = 0.359) and CS discrimination (p < 0.001) for SCR, and main effect of stimulus F(1,52) = 397.662, p < 0.001, ηp^2^ = 0.884) with CS discrimination (p < 0.001) for RR. No significant interactions or main effect of group were found.

During FE training, a main effect of stimulus (F(2,104) = 39.281, p < 0.001, ηp^2^ = 0.430) evidenced successful recall of acquisition 24h after. A significant Stimulus∗Block∗Group interaction (F(4,208) = 2.569, p = 0.039, ηp^2^ = 0.047) revealed that only the OCA group showed significant CS discrimination in block 1 (OCA: B1, p = 0.013; B2, p = 0.318; B3, p = 0.929; LF: B1, p = 0.807; B2, p = 0.001; B3, p = 0.247) whereas LF women showed it in block 2, before extinguishing (Supplementary Fig. 14a and b). In SCR, a main effect of stimulus (F(1,51) = 17.951, p < 0.001, ηp^2^ = 0.260) combined with a significant stimulus∗block interaction (F(2,102) = 3.572, p = 0.032, ηp^2^ = 0.065) showed a decline in CS discrimination across blocks (B1, p < 0.001; B2, p = 0.029; B3, p = 0.116), with no group differences. In agreement with previous results, RR showed a main effect of stimulus (F(1,52) = 91.109, p < 0.001, ηp^2^ = 0.637) with significant CS discrimination (p < 0.001). Also a stimulus∗block interaction (F(1.4,71.5) = 112.579, p < 0.001, ηp^2^ = 0.684) emerged, with CS discrimination decreasing across blocks (B1, p < 0.001; B2, p < 0.001; B3, p = 0.046), with no differences between groups. These results suggest similar patterns of FA and within-session extinction between OCA users and LF women, supporting the idea that synthetic and endogenous estrogens exert comparable effects on fear learning.

### 4.2 Vaginal cytology analysis

A novel method for classifying women in menstrual cycle phases was introduced based on Papanicolaou's reports of varying vaginal epithelium phenomena across the menstrual cycle (1933). When studying the differences between the classification according to Bull et al. (2019), and the classification performed following vaginal cytology evaluation, significant differences were seen (p < 0.001, Fisher's exact test). 80% of the EF samples, 52.6% of the LF samples, and 76.9% of the ML samples were correctly classified, leaving LF as the phase with the lowest accuracy in classification (Supplementary Fig. 15).

## 5 Animal results

### 5.1 Animals’ characteristics

Thirty-two out of 88 mice were male (36.4%). 56 females were divided into 4 groups according to estrous cycle phase on FE day. 11 were in proestrus (12.5%), 15 were in estrus (17.0%), 8 were in metestrus (9.1%), and 22 were in diestrus (25.0%). There were no significant differences among groups for age, pre-extinction sample volume, and days of estrous cycle monitoring. As expected, males were significantly heavier than females across groups (p < 0.001) (Supplementary Table 3).

### 5.2 Effect of hormonal levels on mice

Based on our human results demonstrating the predictive power of the P/E2 ratio for FE, we replicated the paradigm in mice using a two-day cued FA and FE procedure with indirect (estrous cycle phase monitoring) and direct (blood LC-MS) hormonal measurements.

Successful FA and FE patterns were observed across our animal sample. No sex differences were found in either the FA or the FE sessions. Following this result, a group-based analysis was performed where the whole distribution of our sample was accounted for (males and females in all estrous cycle phases), and no significant differences were seen.

The same approach as in the human experiment was followed to explore the effect of directly quantified hormones. First, correlation analyses were performed between hormonal levels and freezing levels across the FE session (For hormone levels across groups, see Table 2). Estradiol levels showed a positive correlation with freezing in three blocks of the task (CS6-10, rs = 0.359, p = 0.001; CS11-15, rs = 0.303, p = 0.004; CS16-20, rs = 0.315, p = 0.003). Progesterone levels also positively correlated with freezing in two blocks of the session (CS11-15, rs = 0.256, p = 0.016; CS26-30, rs = 0.235, p = 0.027). Showing the opposite pattern as individual hormones, the P/E2 ratio negatively correlated with freezing levels in the second block of the task (CS6-10, rs = −0.215, p = 0.044) (Supplementary Fig. 16).

**Table 2:** Mice hormone levels.

	Males (32)	Proestrus (11)	Estrus (15)	Metestrus (8)	Diestrus (22)	*p-value*
Estradiol	1.03 ± 1.13∗∗∗	6.86 ± 3.81	3.60 ± 4.27∗∗	2.95 ± 2.37∗∗	3.39 ± 3.07∗∗	<0.001
Progesterone	552.8 ± 406.5°°°	295.6 ± 100.8°°	1464.0 ± 1536.9°	2097.2 ± 1623.3	3409.6 ± 4706.8	<0.001

Data is presented as mean ± SD. For estradiol ∗ indicates significant differences between the marked group and proestrus (reference group with the highest estradiol) ∗ = p < 0.05, ∗∗ = p < 0.01, ∗∗∗ = p < 0.001. Sample sizes are specified in parentheses next to the group names. For progesterone, ° indicates significant differences between the marked group and diestrus (reference group with the highest progesterone) ° = p < 0.05, °° = p < 0.01. Hormone levels are reported in pg/ml.

Repeated measures ANOVAs were performed, modeling group (High hormone/Low hormone) as a between-subjects factor (see Supplementary Table 4 for low/high hormones group composition by sex and estrous cycle).

No differences in FA or FE learning patterns were seen between low estradiol and high estradiol groups, or between low P/E2 ratio and high P/E2 ratio groups. A group∗CS interaction observed in FA when modeling progesterone group reflected higher freezing on the first CS presentation in the high progesterone group (F(4,344) = 3.446, p = 0.009, ηp^2^ = 0.039). Nevertheless, there were no differences in freezing by the end of the session (p = 0.426), reflecting equal FA. No main or interacting effects of group were seen in the FE session (see Fig. 7 for P/E2 ratio behavioral data, Supplementary Fig. 17 for estradiol behavioral data, and Supplementary Fig. 18 for progesterone behavioral data).

**Fig. 6:**
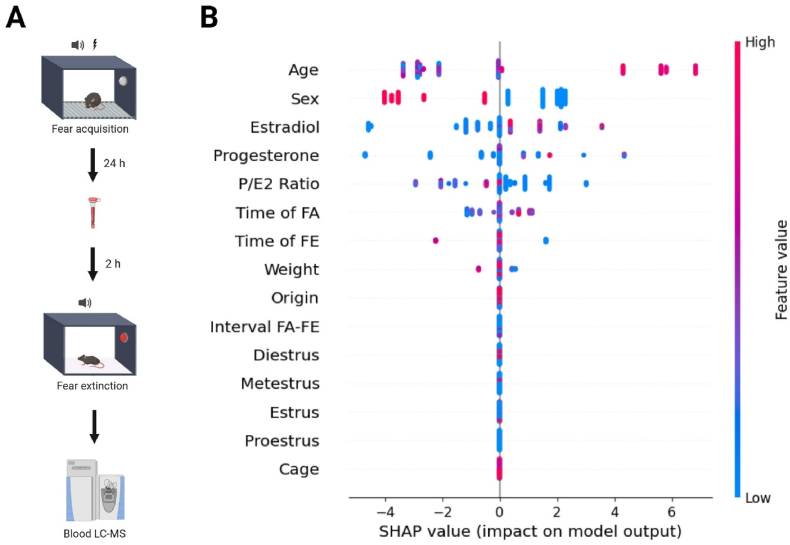
SHAP summary plot showing which features influence the model prediction the most to predict fear extinction in both male and female mice, measured using freezing percentage. Panel A shows a schematic representation of the experimental design, and Panel B shows the HGBRT outcome. The higher the SHAP, the higher the probability of a higher amount of time spent freezing during fear extinction (FE) training. Features are first sorted by their global impact (y-axis). For each individual in the sample, a dot represents the attribution value for each feature from low (blue) to high (red). FA = fear acquisition.

**Machine learning analysis.** Following the same approach as in our human data, we moved forward with a HGBRT to further explore the effects of hormonal variables on FE (target variable defined as the average freezing in the FE session). The model achieved a mean Absolute Error (MAE) of 13.03 with a standard deviation (SD) of 2.77. The mean Root Mean Square Error (RMSE) was 15.18 (SD = 2.94) and the mean R2 was 0.015 (SD of 0.23). The P/E2 ratio ranked among the top 5 most predictive variables, together with estradiol and progesterone. In the same line as the human results, a negative relationship between the P/E2 Ratio and freezing during FE was displayed (Fig. 6) (For SHAP dependence plot see Supplementary Fig. 19).

**Fig. 7:**
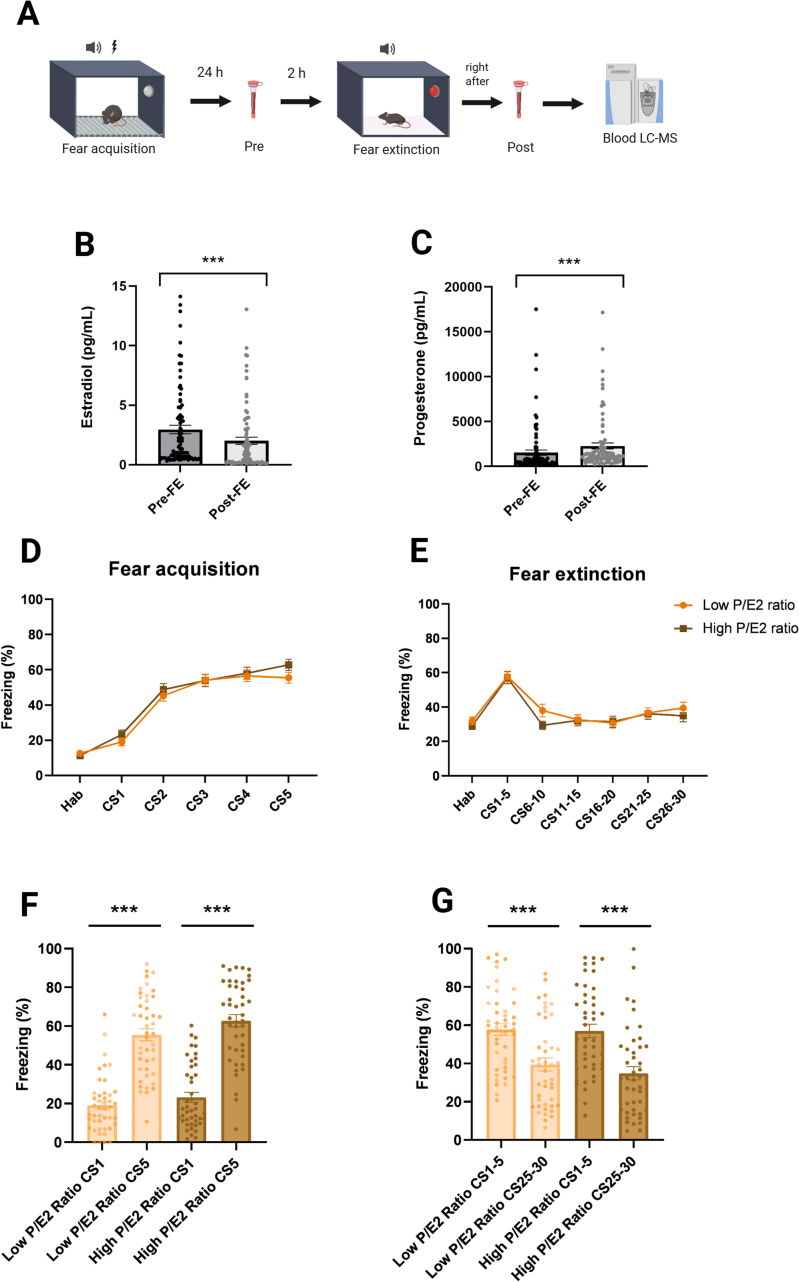
Change in pre-extinction and post-extinction mouse blood progesterone and estradiol levels, and analyses by progesterone/estradiol (P/E2) ratios for fear acquisition and extinction Panel A shows a schematic representation of the experimental design, Panel B shows the change in progesterone levels before and after fear extinction, Panel C shows the change in estradiol levels before and after fear extinction, Panel D shows fear acquisition, Panel E shows fear extinction, Panel F shows comparison of early and late timepoints in fear acquisition, and Panel G shows a comparison of early and late timepoints in fear extinction. Hab: habituation, CS1, CS2, CS3, CS4, CS5: conditioned stimulus, CS1-5, CS6-10, CS11-15, CS16-20, CS21-25, C26-30: blocks grouping 5 conditioned stimuli each. P/E2 ratios used in Panels D to G were calculated from Pre samples.

### 5.3 Effect of stress on sex steroid levels

Two blood samples were obtained from our mice: the first one (pre) was collected 2 h before FE, and the second one (post) was collected right after FE. Based on previous reports of changes in estradiol and progesterone levels after stress, we explored whether undergoing FE would also trigger changes in these hormones’ levels. Significant differences in estradiol levels were found between pre and post-FE samples, showing a FE-mediated decrease in estradiol levels (t(82) = 5.171, p < 0.001). Conversely, the opposite pattern was seen in progesterone levels, as this hormone showed an increase in its levels after the FE session (t(83) = -5.162, p < 0.001) (Fig. 7).

## 6 Discussion

This study explored the effects of menstrual and estrous cycle phases and sex hormone fluctuations on FA and within-session FE in both humans and mice. It integrates direct hormone measurements (LC/MS steroid quantification) and indirect assessments (menstrual/estrous cycle phase allocation). Machine learning analyses showed that the P/E2 ratio proves to be one of the main predictive variables for FE in both species, with high ratios predicting enhanced FE. Overall, these results suggest a novel cross-species interplay between progesterone and estradiol in modulating fear processing, highlighting the importance of considering sex and sex-related variables when studying memory processes.

Currently, a consensus has not been reached in the literature focused on the effect of estradiol on fear memory. Animal studies have often reported estradiol to be related to improvements in different types of learning, including spatial memory, working memory, object recognition, and both cued and contextual fear conditioning, among others (Finney et al., 2020; Hampson and Morley, 2013; Patel et al., 2022). However, various studies have reported the opposite effect (Galea et al., 2001; Holmes et al., 2002; Imwalle et al., 2006). When focusing on fear memory, human studies point towards an association between high estradiol and enhanced FE recall, but these same studies show on many occasions no significant role of estradiol on within-session extinction (Graham and Milad, 2013; Milad et al., 2010; White and Graham, 2016). Oppositely, other studies report improved within-session extinction in high estradiol conditions (Kaczmarczyk et al., 2024; Wegerer et al., 2014). In addition, studies taking trauma into account highlight the facilitating effect of estradiol on within-session FE in women with PTSD (Glover et al., 2012).

In this study, when comparing our five human groups, we did not find a significant influence of group. However, when focusing our analyses on EF (low estradiol) and LF (high estradiol) women, a near-significant interaction suggests that, unlike LF women, EF participants do not seem to extinguish fear responses. The same pattern is seen with LC-MS quantified estradiol levels, where low estradiol participants exhibit impaired FE learning and still show significant CS discrimination by the end of the session. Conversely, high estradiol participants were able to fully extinguish the association. In our animal experiment, similarly to in our human data, no differences were seen when comparing our five groups’ FE training. When focusing on LC-MS quantified hormone levels, no individual or combined effect of estradiol or progesterone was seen.

When focusing on progesterone, its role in fear conditioning remains inconsistent across studies. Different publications show no significant effect of progesterone on FE in humans or mice (Kaczmarczyk et al., 2024; Milad et al., 2010; Zeidan et al., 2011). Complementary, Pineles et al. (2016) found impaired extinction recall in women with PTSD during the ML menstrual phase, suggesting a potential interaction between hormonal levels and trauma. In concordance with the literature, our results show no significant influence of progesterone on FA or FE, in humans or mice. Nevertheless, exploratory observations of the data suggest possible FE impairments in ML women (characterized by high progesterone), as seen by a lack of CS discrimination across both FA and FE phases. This observation may point to a subtle, multifactorial influence of the menstrual cycle on fear learning that extends beyond hormonal influence. Further investigation is required to substantiate this hypothesis.

Results across studies hint at a potential combined effect of estradiol and progesterone (Kaczmarczyk et al., 2024; Milad et al., 2009). Therefore, a combined approach seems to be optimal for further understanding the hormonal modulation of fear learning. The progesterone to estradiol ratio provides a measure of the relationship between the two steroids in each individual, accounting for their independent fluctuations across the menstrual cycle (Seligowski et al., 2020). Milad et al. (2010) studied the effect of the P/E2 ratio on FE recall through a median split and correlational analysis, but found no significant results. In this same line, we found no significant role of the P/E2 ratio when comparing high and low P/E2 ratios in humans or mice. Nevertheless, when performing analyses of higher complexity, we were able to show the relevance of the P/E2 ratio through a predictive machine learning approach. Thus, these findings suggest that the P/E2 ratio should be understood not as a simple predictor, but as a variable whose relevance to fear extinction is best revealed through analytical tools capable of capturing non-linear relationships and interactions.

In our HGBRT, the P/E2 ratio ranked as one of the top importance variables for FE in both species. In humans, high P/E2 ratios showed a negative impact on SCR CS discrimination during FE, suggesting that higher P/E2 ratios are associated with enhanced extinction learning. This model further revealed a complex interplay of multiple hormones along with personality characteristics such as trait anxiety. In the animal experiment, the evidence for the P/E2 ratio rests on three complementary findings that converge on the same conclusion. First, the P/E2 ratio was negatively correlated with freezing during the second block of extinction (CS6–10, rs = −0.215, p = 0.044), indicating that higher ratios were associated with less freezing and therefore better extinction. Second, the HGBRT ranked the P/E2 ratio among the top five predictors of average freezing during FE, alongside estradiol and progesterone individually. Third, the extinction session itself produced a significant post-FE increase in the P/E2 ratio, driven by opposing shifts in progesterone (increase) and estradiol (decrease), which is consistent with the ratio being a neuroendocrine correlate of the extinction process rather than merely a passive baseline variable. The main difference between the human and animal results is the higher ranking of individual progesterone and estradiol levels as predictive variables in the animal experiment, which may suggest species differences in hypothalamic-pituitary-gonadal (HPG) axis functioning during FE. Overall, these results consistently point to higher P/E2 ratios as a correlate of enhanced FE across both species, supporting the relevance of the P/E2 ratio as a candidate biomarker of fear extinction, but requiring further investigation with larger samples.

Stress exposure is known to alter hormonal states, but contradictory results are found in the literature. The activation of the HPG axis stimulates progesterone release, and therefore, its levels are known to increase after stress (Deis et al., 1989; Herrera et al., 2016; Wirth et al., 2007). Conversely, estradiol's dynamics under stress are not clear, since contradicting results are found in the literature (e.g. Pletzer et al., 2021;Roney and Simmons, 2015;Schliep et al., 2015;Shors et al., 1999). Despite fear acquisition and extinction not being considered stressors per se, fear conditioning processes have been reported to elicit physiological responses similar to those shown under stress (Swiercz et al., 2018). When comparing pre- and post-extinction hormone levels in mice, a progesterone increase is observed, which is consistent with the stress literature. On the contrary, the opposite pattern is seen in estradiol, where its levels in blood decrease after FE training. This differential shift translates into an increase in the P/E2 ratio, which, together with our machine learning results, could be understood as a neuroendocrine feature of FE.

In addition to the effects of endogenous hormones, it is also crucial to examine the influence of exogenous hormones on fear learning, particularly those in OCAs. Previous research has shown that OCAs can enhance FA and FE recall (Bartholomew et al., 2022; Lonsdorf et al., 2015), while different studies have reported opposite results, relating OCAs to impaired FE recall (Graham and Milad, 2013; White and Graham, 2016). We expected to find similar FA and within-session FE patterns in the OCA and LF human groups, due to high levels of synthetic and natural estrogens, respectively. Despite some differences in late FA and early FE, both groups showed successful FA and within-session FE, supporting the idea that natural and synthetic hormones have similar effects on FE training, as previously reported (Graham and Milad, 2013; Lonsdorf et al., 2015).

To assess the menstrual cycle phase indirectly, we classified participants into EF, LF, and ML groups through vaginal cytologies, following Papanicolaou's reports of different cellular profiles in the vaginal epithelium across the cycle (Papanicolaou, 1933). However, this classification showed limited accuracy, specifically for the LF group (52.6% accuracy), compared to higher accuracy in EF (80% accuracy) and ML (76.9% accuracy). This inaccuracy might result from phase overlaps, as participants tested near LF limits may either still exhibit EF characteristics or begin showing ML features.

Caution should be taken when interpreting these results, since this study includes data from two different species, and hormonal quantification from different media (saliva in humans and blood in mice). Nevertheless, despite these differences, similar findings are obtained across species, which highlights the robustness and translatability of these results. Among the limitations of this study, we find the inclusion of exclusively healthy participants, limiting the generalizability of results to the clinical population. In addition, participants in the OCA group were prescribed various oral contraceptives with different progestin types and concentrations. This treatment heterogeneity should be considered when interpreting our results. Moreover, different sensory modalities and stimulus numbers were used in the experimental paradigms, as humans were presented with visual CS+ and CS-, while mice were presented with a single auditory CS.

In conclusion, this study highlights the role of the progesterone and estradiol ratio in modulating FE. Our findings suggest that the previously reported positive effects of estradiol on FE recall may not be limited to extinction memory consolidation. Instead, estradiol seems to already show facilitating effects during FE training in humans, with this effect being strengthened by the combined influence of estradiol and progesterone. This finding was replicated in an experiment conducted with mice, further supporting the importance of the progesterone and estradiol interplay in FE, as well as reinforcing the translatability of our findings. Overall, these findings fill a critical gap in the literature and highlight the complex modulation of hormonal interactions on fear memory across species. This P/E2 ratio could become an integrating factor in memory research, helping to reduce variability between studies, improving the comparison of results, and ultimately contributing to more consistent and robust findings across both human and animal research.

## CRediT authorship contribution statement

**Jaime F. Nabás:** Conceptualization, Data curation, Formal analysis, Investigation, Methodology, Writing – original draft, Writing – review & editing. **Eric R. Velasco:** Conceptualization, Data curation, Formal analysis, Investigation, Methodology. **David Fabregat-Safont:** Investigation. **Élida Alechaga:** Investigation. **Alex Gomez-Gomez:** Investigation. **Marta Torrent:** Investigation. **Mariana G. Fronza:** Writing – review & editing. **Victoria Mueller:** Formal analysis. **Mohammed R. Milad:** Writing – review & editing. **Rafael Torrubia:** Conceptualization, Writing – review & editing. **Miquel A. Fullana:** Conceptualization, Writing – review & editing. **Katharina Schultebraucks:** Formal analysis, Writing – review & editing. **Oscar Pozo:** Investigation, Writing – review & editing. **Raul Andero:** Conceptualization, Data curation, Funding acquisition, Methodology, Project administration, Supervision, Writing – original draft, Writing – review & editing.

## Declaration of competing interest

The authors declare the following financial interests/personal relationships which may be considered as potential competing interests: Raül Andero's role as Editorial Board Member, had no involvement in the peer review of this article and had no access to information regarding its peer review. Full responsibility for the editorial process for this article was delegated to another journal editor. If there are other authors, they declare that they have no known competing financial interests or personal relationships that could have appeared to influence the work reported in this paper.

## Data Availability

Data will be made available upon reasonable request.
