# Supplementary material for: The progesterone to estradiol ratio predicts fear extinction in mice and humans
Source: Neurobiol Stress. 2026 May 22;43:100823. doi: 10.1016/j.ynstr.2026.100823 (PMC13273471; doi:10.1016/j.ynstr.2026.100823)
Supplement: Multimedia component 1 [file mmc1.docx]

**
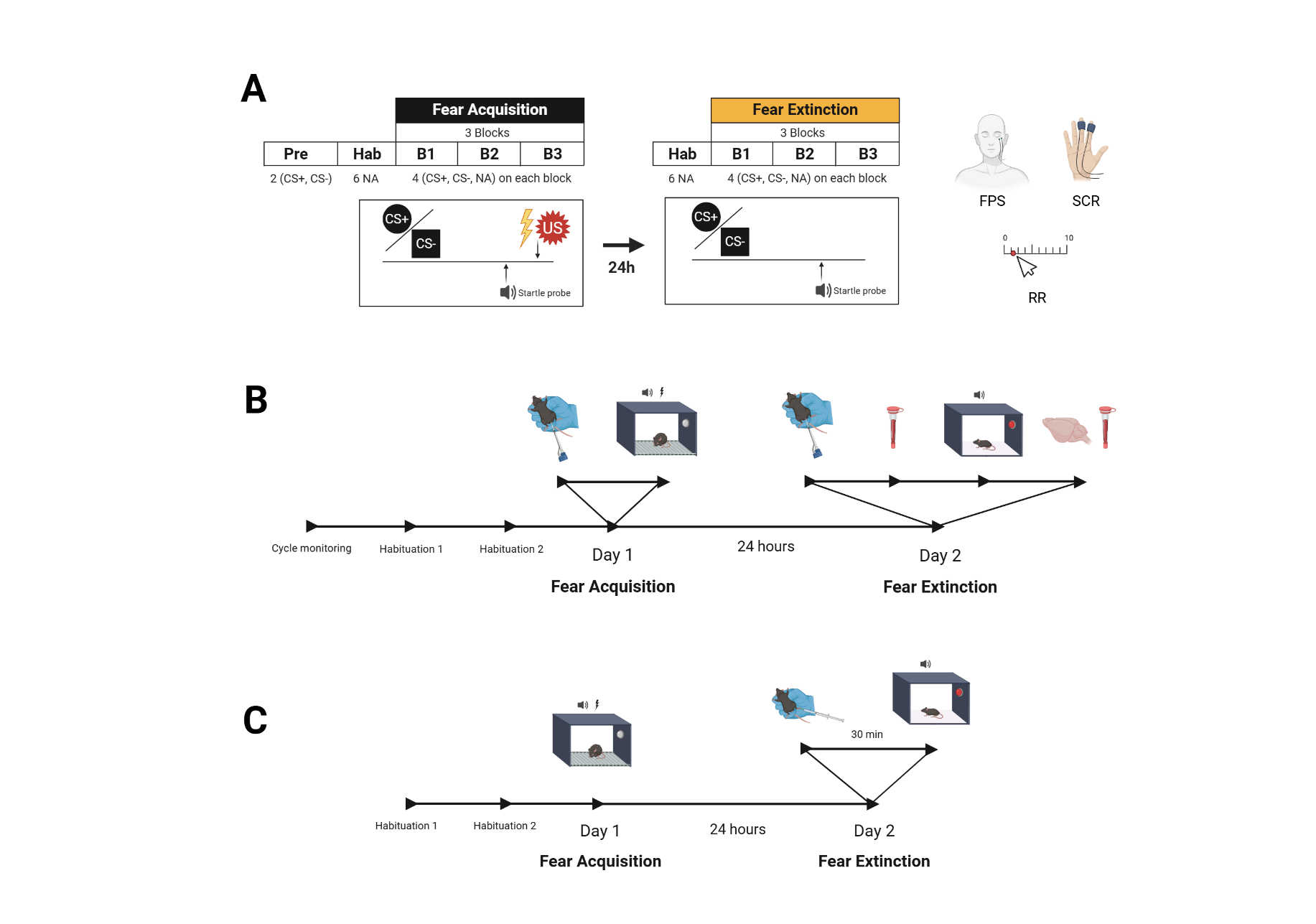
Supplementary Figure 1.** **Schematic representation of the two-day fear acquisition and fear extinction paradigms used in the human and animal studies.** Panel A shows the human experiment, where day 1 consisted of a pre-acquisition phase (2 unreinforced CS+/CS- presentations), followed by a habituation phase (6 NA), and a FA phase that was divided into 3 blocks (B1, B2, B3). Each block consisted of 4 presentations of each stimulus (4 CS+/ CS-/ NA). Day 2 started with a habituation phase (6 NA), followed by a FE phase divided into 3 blocks with the same structure as day 1. No US was administered during FE. B1, B2, B3: block, CS+: reinforced CS, CS-: unreinforced CS, Hab: habituation phase, NA: noise alone, Pre: pre-acquisition phase, US: unconditioned stimulus. Panel B shows the endogenous hormones animal experiment, where one blood sample was extracted before FE training, and another after.
