# Supplementary material for: The progesterone to estradiol ratio predicts fear extinction in mice and humans
Source: Neurobiol Stress. 2026 May 22;43:100823. doi: 10.1016/j.ynstr.2026.100823 (PMC13273471; doi:10.1016/j.ynstr.2026.100823)
Supplement: Multimedia component 2 [file mmc2.docx]

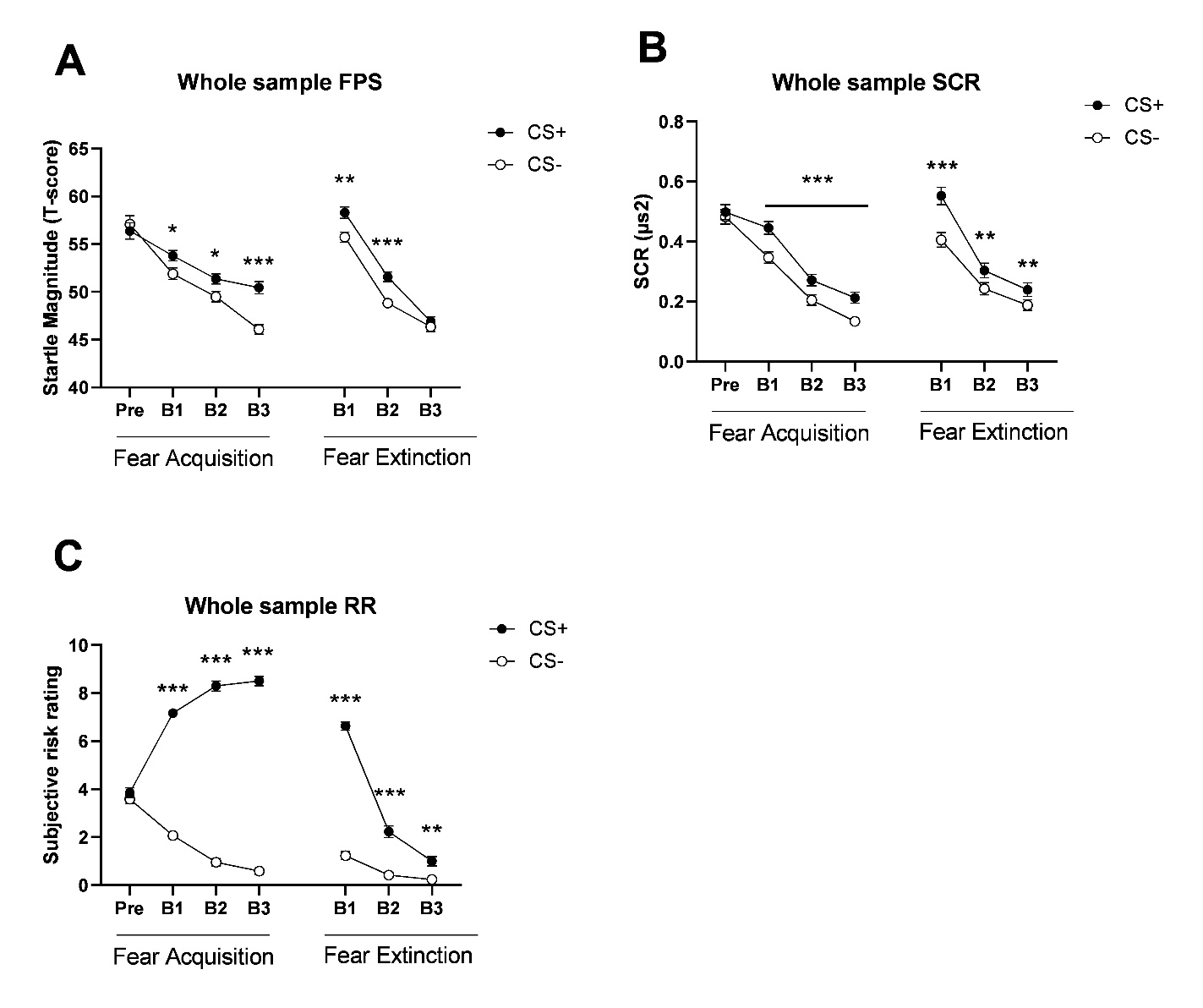


**Supplementary Figure 2. Whole sample analyses for fear acquisition and extinction in humans.** Panel A shows fear potentiated startle, Panel B shows skin conductance response, Panel C shows subjective risk ratings. Pre: pre-acquisition trials, B1,B2,B3: block, CS+: reinforced CS, CS-: non-reinforced CS, NA: noise alone. * = p<0.05, ** = p<0.01, *** = p<0.001. Horizontal line above blocks indicates main effect stimulus.
