# Supplementary material for: The progesterone to estradiol ratio predicts fear extinction in mice and humans
Source: Neurobiol Stress. 2026 May 22;43:100823. doi: 10.1016/j.ynstr.2026.100823 (PMC13273471; doi:10.1016/j.ynstr.2026.100823)
Supplement: Multimedia component 4 [file mmc4.docx]

**
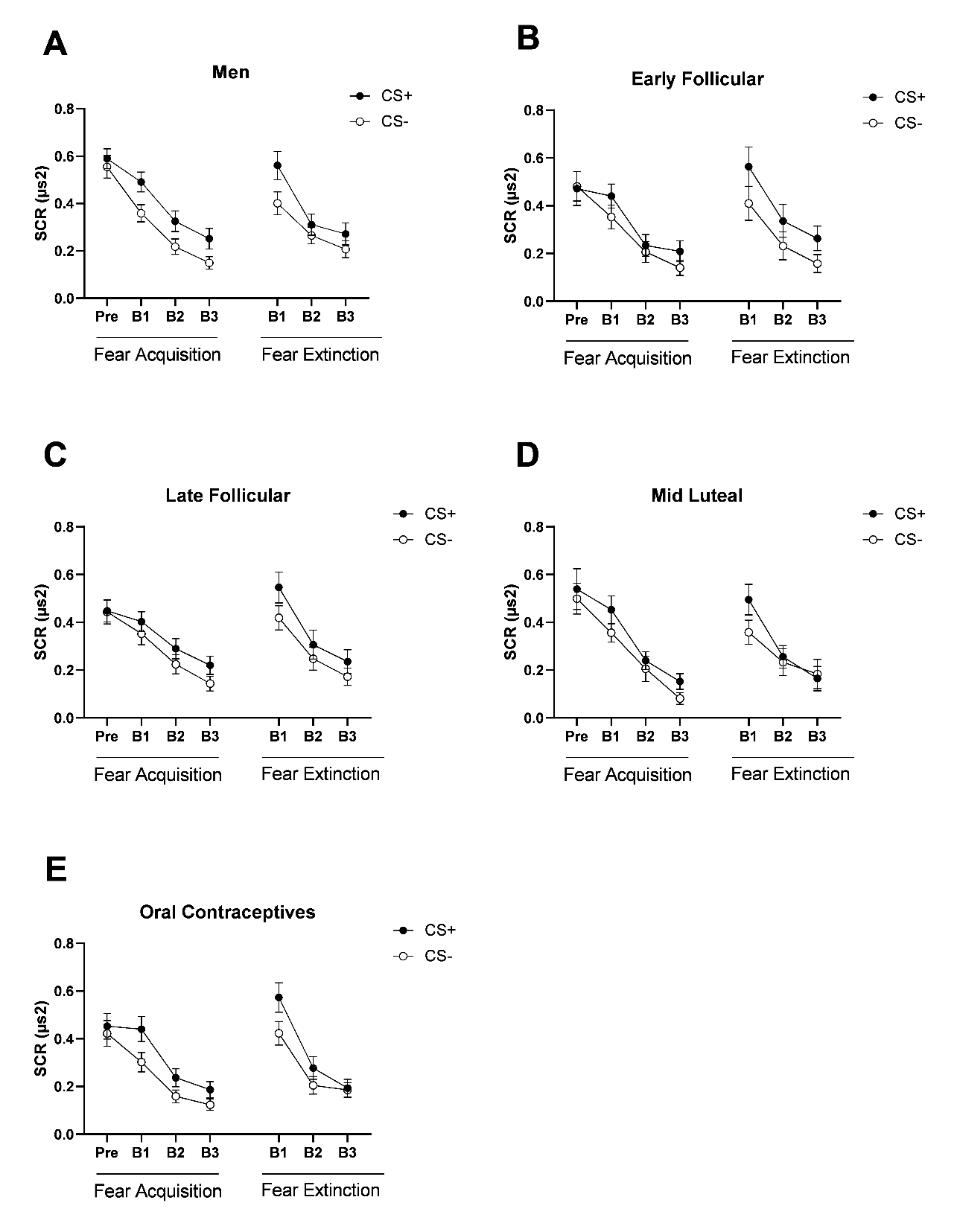
Supplementary Figure 4. Group analyses by sex and menstrual cycle phase for fear acquisition and extinction in humans. Skin conductance response.** Panel A shows men, Panel B shows early follicular women, Panel C shows late follicular women, Panel D shows mid-luteal women, and Panel E shows women taking oral contraceptives. Pre: pre-acquisition trials, B1,B2,B3: block, CS+: reinforced CS, CS-: non-reinforced CS.
