# Supplementary material for: The progesterone to estradiol ratio predicts fear extinction in mice and humans
Source: Neurobiol Stress. 2026 May 22;43:100823. doi: 10.1016/j.ynstr.2026.100823 (PMC13273471; doi:10.1016/j.ynstr.2026.100823)
Supplement: Multimedia component 6 [file mmc6.docx]

**
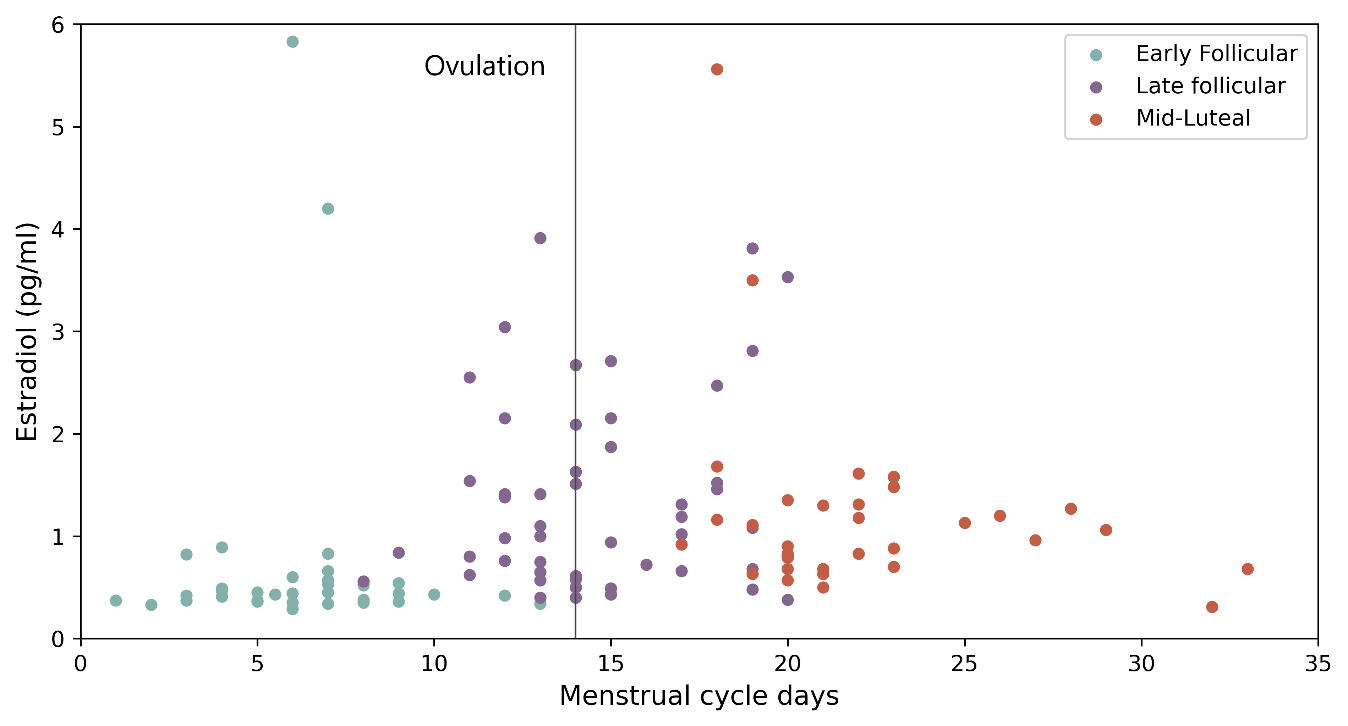
Supplementary Figure 6. Human estradiol levels throughout the menstrual cycle.** The Y axis represents estradiol levels in saliva, and the X axis represents the days of the menstrual cycle in which the saliva samples were collected. Blue dots represent participants in the early follicular phase, purple dots represent subjects in the late follicular phase, and red dots represent participants in the mid-luteal phase.
