# Supplementary material for: The progesterone to estradiol ratio predicts fear extinction in mice and humans
Source: Neurobiol Stress. 2026 May 22;43:100823. doi: 10.1016/j.ynstr.2026.100823 (PMC13273471; doi:10.1016/j.ynstr.2026.100823)
Supplement: Multimedia component 9 [file mmc9.docx]

**
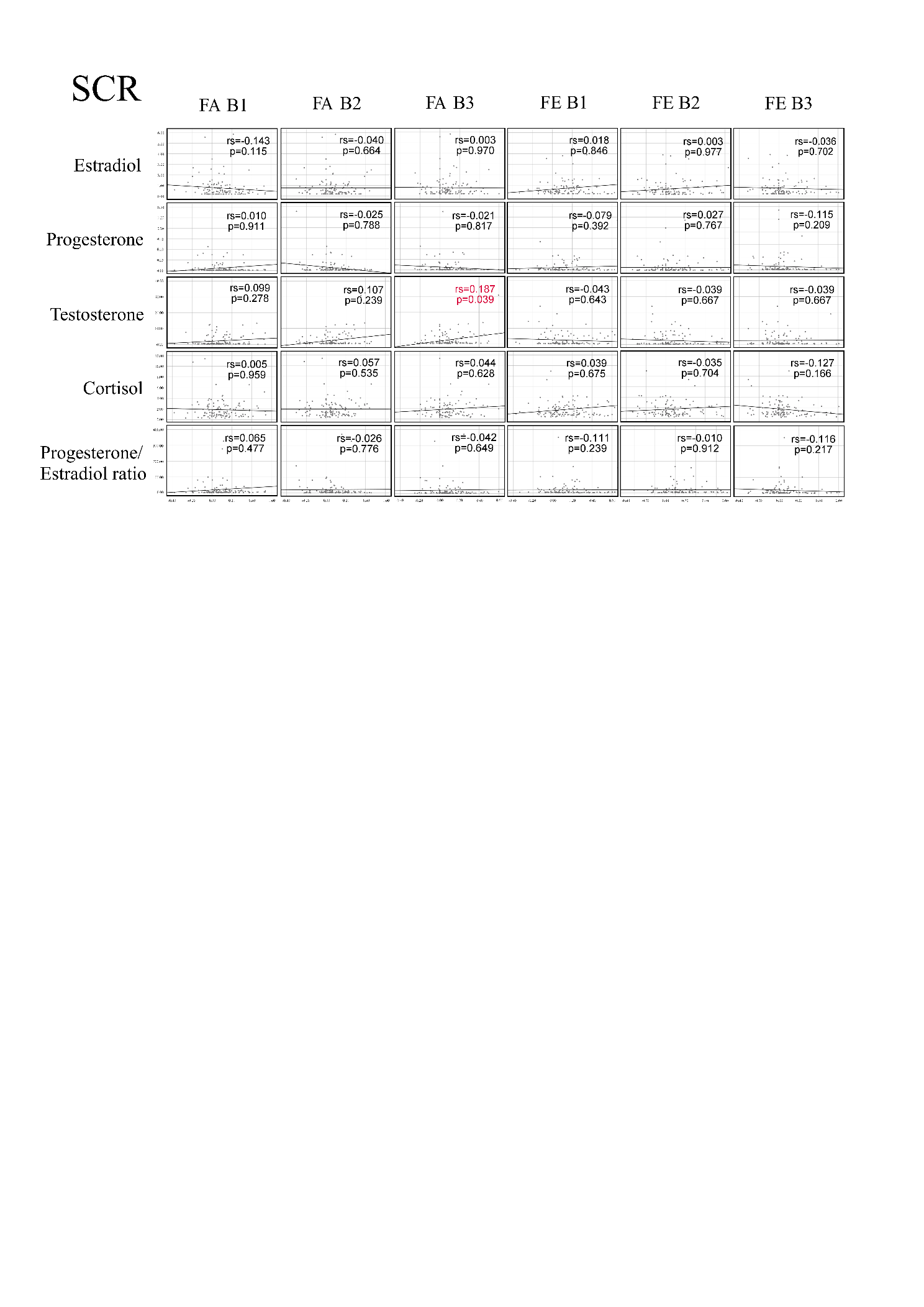
Supplementary Figure 9. Correlations between hormone levels and fear acquisition and extinction in humans. Skin conductance response.** On the Y axis of the matrix are the different hormones analyzed. On the X axis, the different blocks of the task are represented. FA: fear acquisition, FE: fear extinction, B1, B2, B3: block, rs: Spearman’s correlation coefficient. Estradiol is reported as pg/ml, progesterone, testosterone, and cortisol as ng/ml. Red text indicates statistical significance.
