# Supplementary material for: The progesterone to estradiol ratio predicts fear extinction in mice and humans
Source: Neurobiol Stress. 2026 May 22;43:100823. doi: 10.1016/j.ynstr.2026.100823 (PMC13273471; doi:10.1016/j.ynstr.2026.100823)
Supplement: Multimedia component 11 [file mmc11.docx]

**
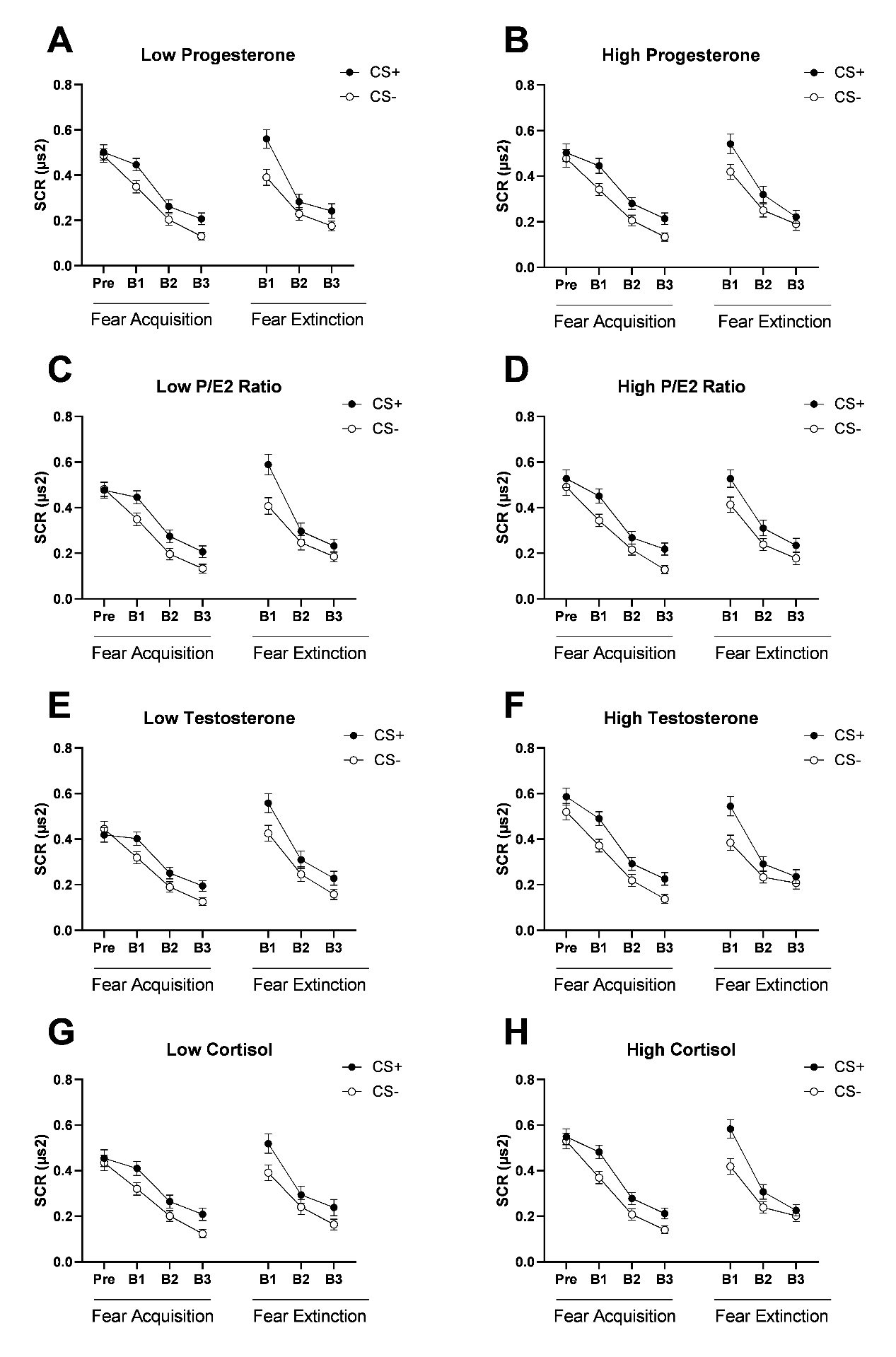
Supplementary Figure 11. Analyses by hormonal levels for fear acquisition and extinction in humans. Skin conductance response.** Panel A shows low progesterone participants, Panel B shows high progesterone participants, Panel C shows low P/E2 ratio participants, Panel D shows high P/E2 ratio participants, Panel E shows low testosterone participants, and Panel F shows high testosterone participants, Panel G shows low cortisol participants, and Panel H shows high cortisol participants, . Pre: pre-acquisition trials, B1,B2,B3: block, CS+: reinforced CS, CS-: non-reinforced CS.
