# Supplementary material for: The progesterone to estradiol ratio predicts fear extinction in mice and humans
Source: Neurobiol Stress. 2026 May 22;43:100823. doi: 10.1016/j.ynstr.2026.100823 (PMC13273471; doi:10.1016/j.ynstr.2026.100823)
Supplement: Multimedia component 13 [file mmc13.docx]

**
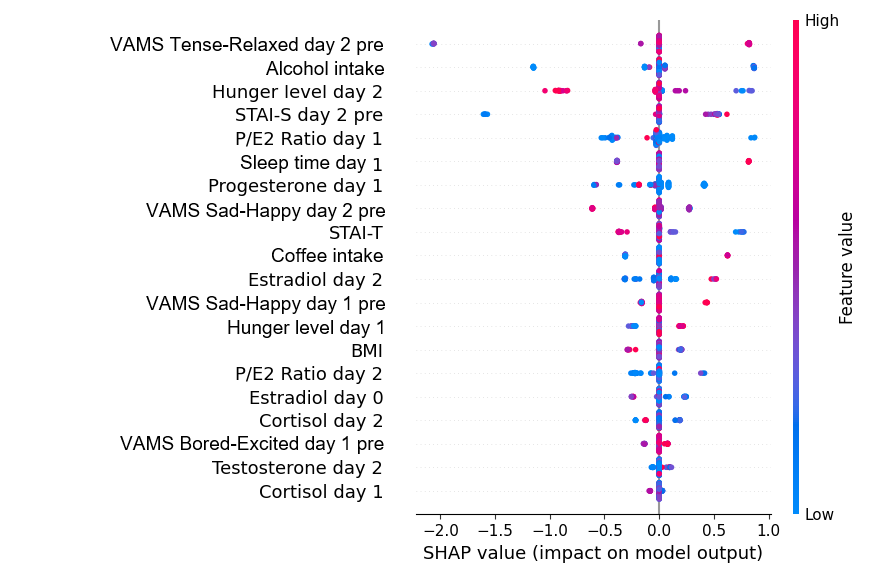
Supplementary Figure 13.** **SHAP summary plot showing which features influence the model prediction the most to predict fear extinction measured using fear potentiated startle in humans.** The higher the SHAP, the higher the probability of higher CS discrimination. Features are first sorted by their global impact (y-axis). For each individual in the sample, a dot represents the attribution value for each feature from low (blue) to high (red). Features are first sorted by their global impact (y-axis). For each individual in the sample, a dot represents the attribution value for each feature from low (blue) to high (red).
