# Supplementary material for: The progesterone to estradiol ratio predicts fear extinction in mice and humans
Source: Neurobiol Stress. 2026 May 22;43:100823. doi: 10.1016/j.ynstr.2026.100823 (PMC13273471; doi:10.1016/j.ynstr.2026.100823)
Supplement: Multimedia component 14 [file mmc14.docx]

**
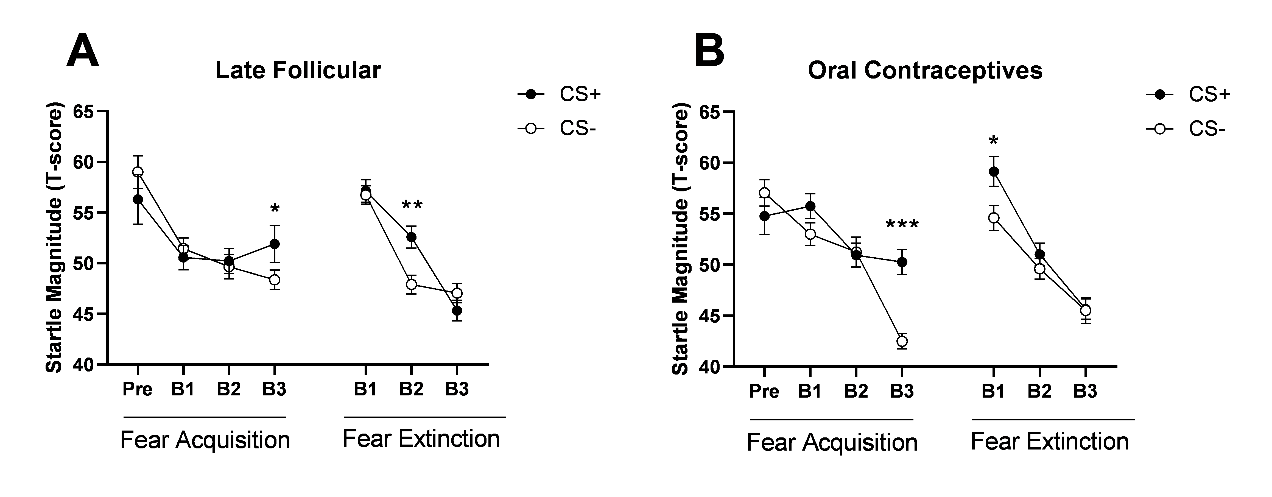
Supplementary Figure 14. Fear acquisition and extinction in late follicular and** **oral contraceptives human groups.** **Fear-potentiated startle.** Panel A shows the late follicular group, and Panel B shows the oral contraceptives group. B1, B2, B3: block, CS+: reinforced CS, CS-: non-reinforced CS, pre: pre-acquisition trials. * Indicates CS discrimination (e.g., higher responses to CS+>CS-). * = p<0.05, ** = p<0.01, *** = p<0.001.
