# Supplementary material for: The progesterone to estradiol ratio predicts fear extinction in mice and humans
Source: Neurobiol Stress. 2026 May 22;43:100823. doi: 10.1016/j.ynstr.2026.100823 (PMC13273471; doi:10.1016/j.ynstr.2026.100823)
Supplement: Multimedia component 15 [file mmc15.docx]

**
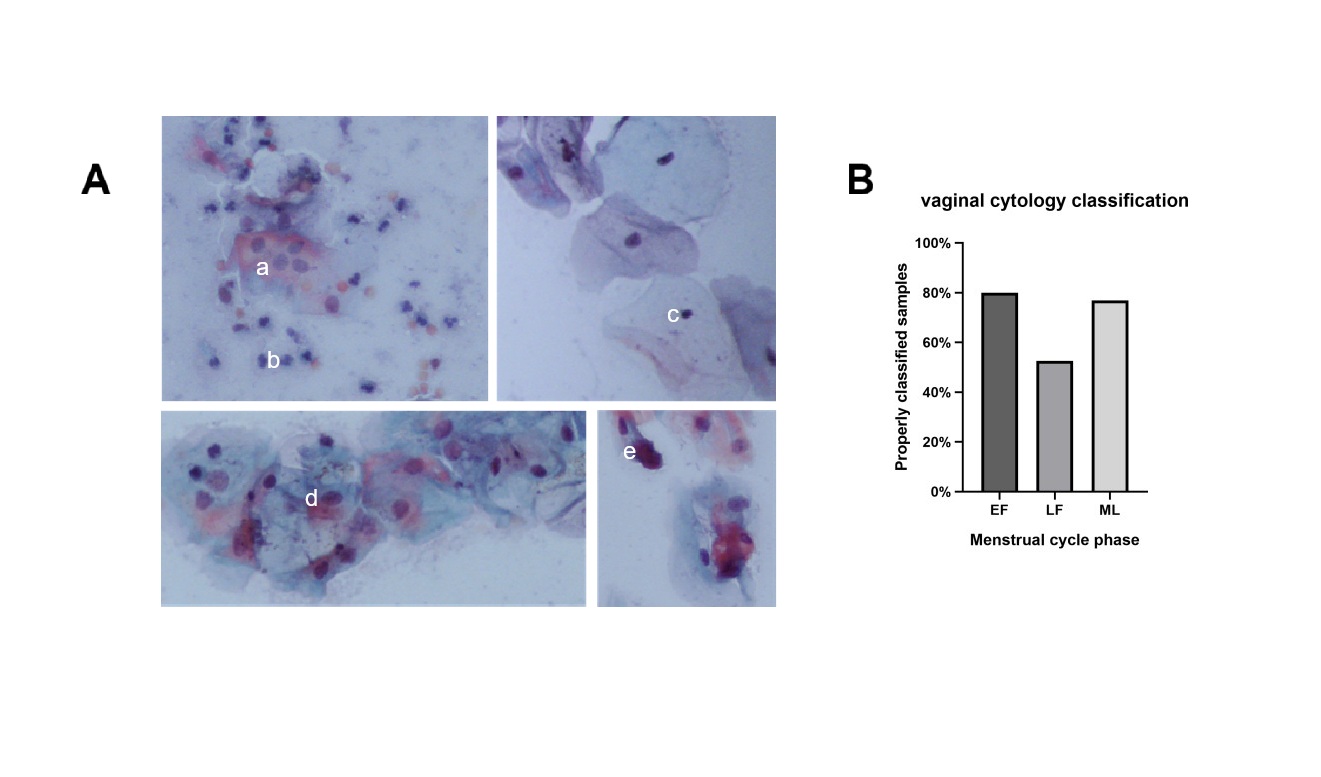
Supplementary Figure 15. Accuracy of human vaginal cytology classification.** Panel A brightfield microscopy images show defining characteristics in vaginal cytology classification, including intermediate cells (a), leukocytes (b), superficial cells (c), intermediate cells with cytoplasmic granulations (d), and erythrocytes (e). Panel B shows the percentages of properly classified vaginal cytologies according to the phase of the menstrual cycle.
