# Supplementary material for: The progesterone to estradiol ratio predicts fear extinction in mice and humans
Source: Neurobiol Stress. 2026 May 22;43:100823. doi: 10.1016/j.ynstr.2026.100823 (PMC13273471; doi:10.1016/j.ynstr.2026.100823)
Supplement: Multimedia component 16 [file mmc16.docx]

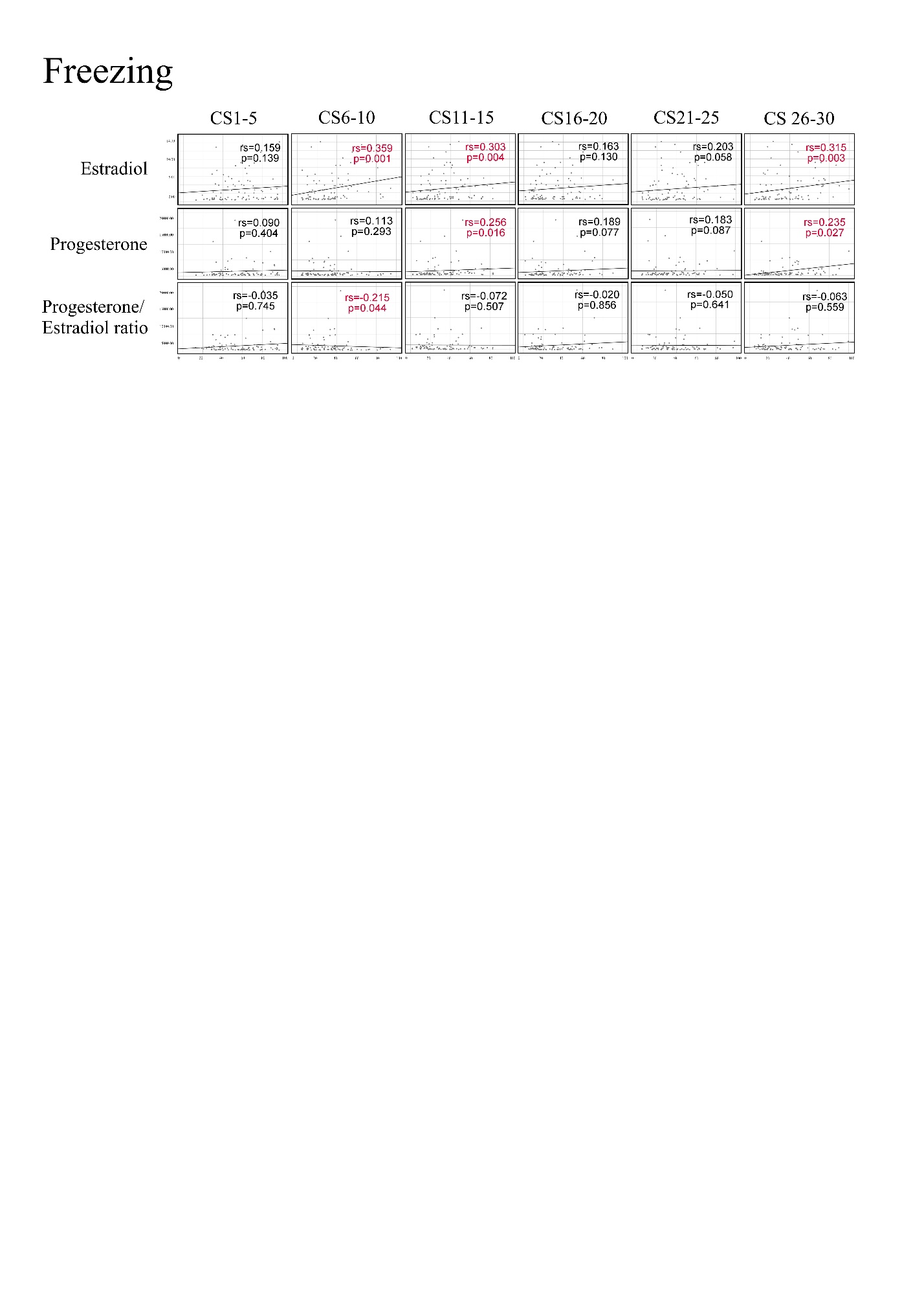
**Supplementary Figure 16. Correlations between hormone levels and freezing levels in fear extinction in mice.** The Y axis of the matrix shows the different hormones analyzed. The X axis shows different blocks. CS1-5, CS6-10, CS11-15, CS16-20, CS21-25, CS26-30: blocks grouping 5 conditioned stimuli each. Estradiol and progesterone are reported as pg/ml. Red text indicates statistical significance.
