# Supplementary material for: The progesterone to estradiol ratio predicts fear extinction in mice and humans
Source: Neurobiol Stress. 2026 May 22;43:100823. doi: 10.1016/j.ynstr.2026.100823 (PMC13273471; doi:10.1016/j.ynstr.2026.100823)
Supplement: Multimedia component 18 [file mmc18.docx]

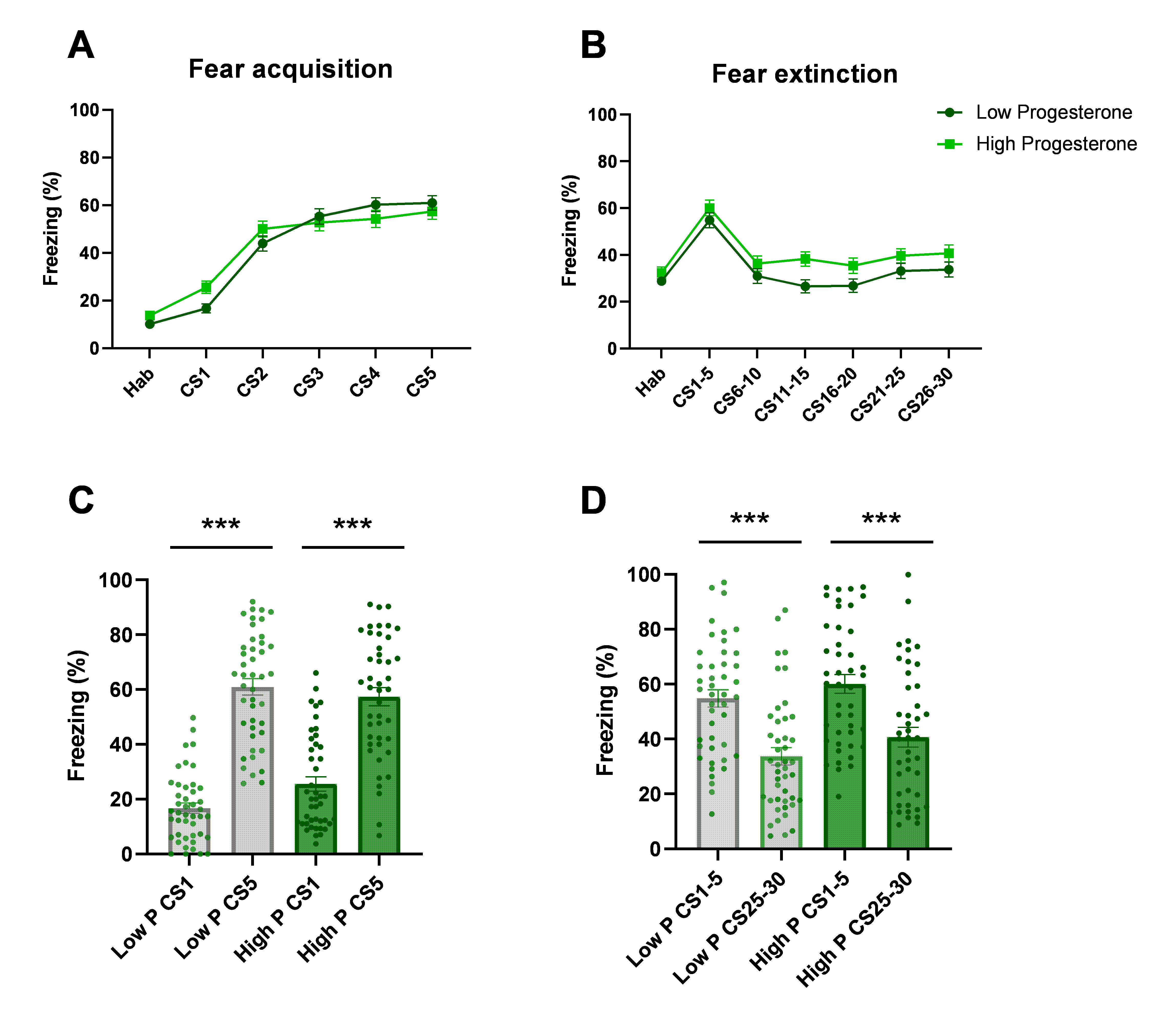
**Supplementary Figure 18. Analyses by progesterone levels for fear acquisition and extinction in mice.** Panel A shows fear acquisition, Panel B shows fear extinction, Panel C shows a comparison of early and late timepoints in fear acquisition, and Panel D shows a comparison of early and late timepoints in fear extinction. Hab: habituation, CS1, CS2, CS3, CS4, CS5: conditioned stimulus, CS1-5, CS6-10, CS11-15, CS16-20, CS21-25, C26-30: blocks grouping 5 conditioned stimuli each.
