# Supplementary material for: The progesterone to estradiol ratio predicts fear extinction in mice and humans
Source: Neurobiol Stress. 2026 May 22;43:100823. doi: 10.1016/j.ynstr.2026.100823 (PMC13273471; doi:10.1016/j.ynstr.2026.100823)
Supplement: Multimedia component 19 [file mmc19.docx]

**
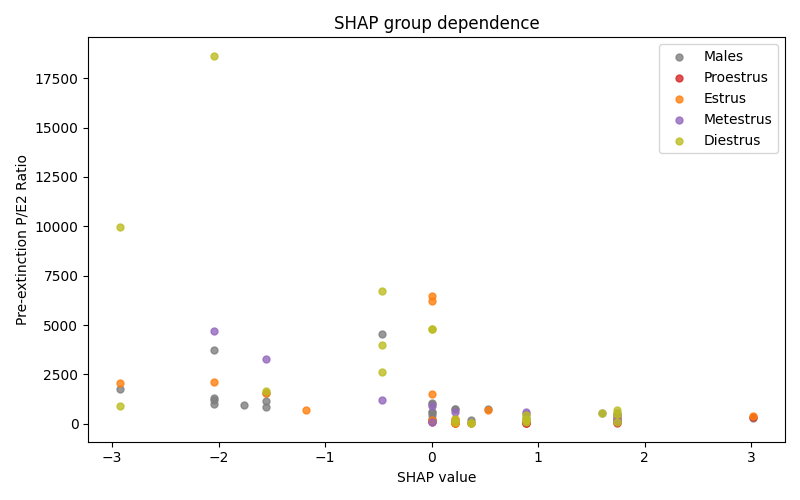
Supplementary Figure 19. SHAP dependence plot in mice.** **Interaction of P/E2 ratio with the different experimental groups.** The distribution of all mice included in the endogenous hormones experiment shows that the majority of animals with high P/E2 ratios are concentrated in the region where negative SHAP values are plotted.
