# Supplementary material for: The progesterone to estradiol ratio predicts fear extinction in mice and humans
Source: Neurobiol Stress. 2026 May 22;43:100823. doi: 10.1016/j.ynstr.2026.100823 (PMC13273471; doi:10.1016/j.ynstr.2026.100823)
Supplement: Multimedia component 20 [file mmc20.docx]

**Supplementary materials and methods**

**Physiological Recordings and Response Definition**

Physiological data were recorded using a BIOPAC MP 150 (BIOPAC Systems Inc., USA) and AcqKnowledge 4.1.0 software.

SCR was recorded by attaching two Ag-AgCl electrodes filled with hydrogel to the middle and index fingers of the non-dominant hand. The signal was sampled at 125 Hz, and SCR magnitudes were computed as the difference between the maximum SCR and the value at response onset, detected in the 1-6 seconds after stimulus onset. Only trials with deflections starting between 1-4 s after stimulus onset were considered valid. When two clearly separated peaks in the SCR were visible, only the maximum SCR response of the first peak was considered. Trials with no response, or magnitudes <0.01 μS, were considered no-response trials and scored as 0. Trials with excessive baseline or artifacts were classified as invalid and discarded.

Startle blink responses were measured by recording the electromyographic activity (EMG) of the orbicularis oculi using two 0.5 cm Ag-AgCl surface electrodes. The raw EMG signal was sampled at 2 kHz and filtered (analog 50-Hz notch filter; and digital infinite impulse response band-pass filter, cut-off frequencies of 28 and 500 Hz), rectified, and smoothed (20-ms moving window average). FPS responses were considered valid if the elevation in the EMG observed after the startle probe started between 20 and 100 ms, with its peak occurring between 20 and 150 ms (33). Startle amplitudes were computed by subtracting the mean response in microvolts (μV) of the 50 ms preceding the startle probe from the response peak value. Trials where no response was detected were scored as 0. Trials with excessive baseline or artifacts were classified as invalid and discarded.

After visual inspection, participants showing invalid SCR responses in 70% of US trials during FA were classified as physiological non-responders (n=1) and excluded from the analyses. Similarly, individuals showing invalid responses in 70% of habituation startle probes were classified as physiological non-responders for FPS (n=2 FA, n=0 FE). Non-responders were identified using raw SCR and raw FPS data. (34).

**Vaginal cytology classification**

Samples were classified based on the following criteria: number of superficial and intermediate cells, presence of endometrial cells, presence of erythrocytes, and presence of cytoplasmic granulations. The EF phase was considered when endometrial cells, erythrocytes, and a predominance of intermediate cells were observed. The LF phase was considered when superficial cells were predominant. The luteal phase was considered when a predominance of intermediate cells was observed, often accompanied by cytoplasmic granulations and an overall loss of well-defined cell shape. 5 participants were discarded from vaginal cytology analyses due to the extremely poor quality of both experimental days' samples.

**Liquid Chromatography – Mass Spectrometry hormonal quantification**

For estradiol determination, 500 µL of saliva were mixed with labeled internal standard and subjected to a liquid-liquid extraction using methyl tert-butyl ether, followed by chemical derivatization with 1,2-dimethylimidazole-5-sulfonyl chloride and a second LLE using hexane. Finally, the organic layer was evaporated and reconstituted in 50 µL of a water:methanol 1:1 mixture. For the determination of Progesterone, Testosterone, Cortisol, Androstendione, Cortisone, 11-dehidrocorticosterone, aHHE, bHHE, 20aDHF, 20bDHF, 5aTHF, 5bTHF, 20aDHE, 20bDHE, 5aTHE, and 5bTHE

steroid hormones, 500 µL of saliva were mixed with labeled internal standard and subjected to an LLE with ethyl acetate, evaporating the organic layer and redissolving the extracts in 100 µL of a water:methanol 1:1 mixture. Extracts were then analyzed by LC-MS/MS using reversed-phase separation with a C18 analytical column, and water and methanol, both with 0.01% formic acid and 1 mM ammonium formiate as mobile phases. Hormones were acquired by selected reaction monitoring (SRM) mode, and their concentrations were determined by external calibration using the ratio between the hormone and the internal standard as a response. Further information regarding method validation, sample treatment conditions, and instrumental details for both methods can be found in the corresponding publications (28,37).

**Estrous cycle classification**

Three different cell types could appear in the preparation: cornified epithelial cells, round nucleated cells, or leukocytes. Proestrus was characterized by a high proportion of nucleated epithelial cells, with very small amounts of cornified cells or leukocytes. Estrus is characterized by the predominance of cornified cells, with few nucleated cells. Metestrus presents a mixture of cornified cells and leukocytes, sometimes also containing some nucleated cells. Diestrus is characterized by a clear predominance of leukocytes, which may include a small proportion of nucleated cells.

**LC-MS/MS mouse blood**

For progesterone quantification, 20 µl of serum was mixed with 20 µl of labeled internal standard solution. After protein precipitation with 100 µl of acetonitrile, samples were centrifuged (3000 × g, 5 min) and the supernatant was transferred to a clean tube. The mixture was vortexed and transferred to a clean tube. Serum samples underwent a liquid-liquid extraction by adding 1 mL of NaCl (saturated solution) and 4 mL of ethyl acetate. Extracts were centrifuged (3000 × g, 5 min), the organic layer transferred into a clean tube, and dried under a nitrogen stream. Dried extracts were reconstituted with 100 µl of methanol and 10 µl were injected into the LC-MS/MS system consisting of an Acquity UPLC system coupled to a triple quadrupole (TQS Micro) mass spectrometer. Steroid detection was performed by selected reaction monitoring (SRM), including two transitions for each analyte. The most specific one was selected for the quantification. Quantification was performed by an external calibration approach using the TargetLynx module of the MassLynx software (Thermo Fisher, Spain).

For estradiol quantification, 100 µl plasma samples were mixed with 20 µl of internal standard (estradiol-d3 at 2 ng/ml) and 1 ml of water and were subjected to two consecutive liquid-liquid extractions with tert-butyl methyl ether. The organic layers were mixed and evaporated under a nitrogen stream. Derivatization with 1,2-Dimethylimidazole-5-sulfonyl chloride was performed at 60 °C for 15 min. Derivatized extracts were evaporated under a nitrogen stream and reconstituted in 1 ml of water. Two additional liquid-liquid extractions with 4 ml of hexane were performed. The organic layers were mixed and evaporated under a nitrogen stream, and the extracts were reconstituted in 100 µl water:methanol (50:50). Ten microliters were injected into the LC-MS/MS system consisting of an Acquity UPLC system coupled to a triple quadrupole (TQS Micro) mass spectrometer. Chromatographic separation was performed in an Acquity BEH C18 column (100 mm × 2.1 mm i.d., 1.7 μm) (Waters Associates) at 55 °C and at a flow rate of 300 μl/ min. Mobile phases consisted of water with ammonium formate (1 mM) and formic acid (0.01 % v/v) and methanol with ammonium formate (1 mM) and formic acid (0.01 % v/v). Quantification of estradiol was performed by SRM by monitoring the transitions of 431 > 96, 431 > 161, and 431 > 367 for estradiol and 434 > 96, 434 > 161, and 434 > 370 for estradiol-d3.

**Extended data analysis**

We normalized the distribution of the SCR data by applying a square root transformation. For analyses including startle responses, we used normalized data (T-scores calculated using all probes for each day). FA and FE training were split into three blocks (B1, B2, B3). Each block consisted of the mean of four startle responses or four skin conductance surges within a stimulus category (CS+, CS-).  For startle data, we calculated both CS+ potentiation (CS+ vs NA) and discrimination (CS+ vs CS-). FA and FE training were analyzed separately for each session and for each measure (FPS, SCR, RR) using repeated measures ANOVA with stimulus (CS+, CS-; and NA for FPS only) and block (B1, B2, B3) as within-subjects factors, and group (M, EF, LF, ML, OC) as between-subjects factor. For specific analyses, the within-subjects group factor was limited to EF and LF, or LF and OCA. Greenhouse-Geiser corrections were used when appropriate. Significant or near-significant interactions (p ≤ 0.1) were followed by pairwise comparisons with Least Significant Difference adjustments. The level of significance was set to p < 0.05 (two-tailed). We report ηp2 as an estimate of effect size. Mean values for age, STAI-T, US intensity, US discomfort, and startle discomfort were compared between groups using one-way ANOVA, and frequencies using Chi-squared tests.

For hormone data, we performed Spearman’s correlations between hormone levels and CS discrimination scores (CS+ – CS-) for both FPS and SCR in each block of the two experimental sessions, and for whole session scores (day 1 hormone levels were correlated with FA, and day 2 hormone levels with FE). Additional repeated measures ANOVA with stimulus (CS+, CS-, ITI for FPS; CS+, CS- for SCR) and block (B1, B2, B3) as within-subjects factors, and group (high hormone and low hormone) as between-subjects factors was performed. Participants were divided into high and low hormone groups by the median split. This calculation was performed by averaging the day 1 and 2 hormone levels for participants with both samples and only the available measure in participants with a lost sample. Progesterone-Estradiol (P/E2) ratios were calculated by dividing progesterone by estradiol levels when both were reported pg/mL, following (40). A Fisher's exact test was performed to study the differences between a cytology-based classification and a menstrual cycle length-based classification. These statistical analyses were performed with IBM SPSS 25.0.

To explore the predictive capacity of different variables over FE, we applied a Histogram-based Gradient Boosting Regression Tree, a variant of traditional Gradient Boosting Machine that utilizes histograms for gradient boosting. This analysis was performed in Python 3.9.21.The primary objective of the analysis was to examine within-session FE. To do so, the outcome variable was the CS discrimination scores in SCR during the FE training session. The predictor variables included demographic and clinical characteristics such as age, sex, Body Mass Index (BMI), sport and smoking habits, coffee and alcohol intake, previous traumatic experience, and if positive, age and number of traumatic experiences, State-Trait Anxiety Inventory (STAI-T and STAI-S), and childhood abuse. Behavior on the day of FA (sleep time, stress level, coffee intake, hunger levels, drug intake), hormone levels at the time of FE training (estradiol, testosterone, progesterone, cortisol, and P/E2 ratio), and group classification (men (M), EF, LF, ML, and OCA) were also included as predictor variables. Data were preprocessed, with categorical variables dummy-coded and numerical values standardized between 0 and 1. Missing values were imputed using k-Nearest Neighbor. Trauma age (TR.Age) was removed due to a high percentage of missingness (65.11%). To guard against overfitting, nested cross-validation was used with 5 inner folds and 5 outer folds. The importance of each variable in predicting the outcome variable was determined using SHapley Additive exPlanation (SHAP) values.

In animal experiments, to check for group differences, we performed repeated measures ANOVA with CS (CS1, CS2, CS3, CS4, CS5) or block (CS1-5, CS6-10, CS11-15, CS16-20, CS21-25, CS26-30) as a within-subjects factor, depending on whether FA or FE were being analyzed. Group (Male, Proestrus, Estrus, Metestrus, Diestrus) was included as a between-subjects factor. Greenhouse-Geiser corrections were used when appropriate. Significant or near-significant interactions (p ≤ 0.1) were followed by pairwise comparisons with Least Significant Difference adjustments. The level of significance was set to p < 0.05 (two-tailed). We report η2 as an estimate of effect size. For hormone data, the same approach as in humans was followed, by first performing Spearman’s correlations between hormone levels and the percentage of time spent freezing during blocks comprising 5 CS each. Repeated measures ANOVAs with block (CS1-5, CS6-10, CS11-15, CS16-20, CS21-25, CS26-30) as a within-subjects factor, and group (high hormone/ratio, low hormone/ratio) as a between-subjects factor were also performed. A Histogram-based Gradient Boosting Regression Tree was used as a predictive model. In this case, the outcome variable studied was the percentage of time spent freezing during the CSs across the FE session

percentage of the CSs spent freezing the FE session. The predictor variables included sex, age, weight, number of mice housed in the same cage, estrous cycle phase (Proestrus, Estrus, Metestrus, Diestrus), hours between FA and FE, time of FA, time of FE, days of estrous cycle monitoring, origin of the mouse (directly from provider or from our colony), volume of plasma extracted before FE, estradiol levels, progesterone levels, and P/E2 ratios.
