# Supplementary material for: The progesterone to estradiol ratio predicts fear extinction in mice and humans
Source: Neurobiol Stress. 2026 May 22;43:100823. doi: 10.1016/j.ynstr.2026.100823 (PMC13273471; doi:10.1016/j.ynstr.2026.100823)
Supplement: Multimedia component 22 [file mmc22.docx]

**SUPPLEMENTARY TABLES**

| Supplementary Table 1. Participant´s characteristics | | | | | | |
| --- | --- | --- | --- | --- | --- | --- |
|  | **Men** | **EF** | **LF** | **ML** | **OCA** | ***p-value*** |
| Age, in years | 24.5 ± 5.3 | 24.0 ± 5.5 | 22.6 ± 3.9 | 24.6 ± 4.7 | 21.6 ± 2.78 | 0.066 |
| STAI-T | 21.5 ± 9.5 | 19.1 ± 8.9 | 21.3 ± 8.4 | 21.7 ± 10.6 | 25.0 ± 9.5 | 0.363 |
| US calibration trials, n | 4.9 ± 2.0 | 3.9 ± 1.1 | 4.1 ± 1.3 | 4.4 ± 1.5 | 3.9 ± 1.3 | 0.096 |
| US intensity, in mA | 4.4 ± 1.9 | 3.8 ± 0.8 | 3.6 ± 1.3 | 3.9 ± 1.3 | 3.5 ± 1.2 | 0.127 |
| US discomfort, 0-9 | 6.9 ± 0.8 | 7.0 ± 0.7 | 7.2 ± 0.8 | 7.2 ± 0.7 | 7.3 ± 0.9 | 0.447 |
| Startle probe discomfort, 0-9 | 6.9 ± 1.8 | 7.1 ± 1.7 | 6.9 ± 1.6 | 6.9 ± 1.6 | 7.4 ± 1.7 | 0.826 |
| Contingency-aware individuals, n (%) | 31 (93.9) | 23 (100) | 27 (90) | 18 (100) | 23 (95.8) | 0.397 |
| \| Supplementary Table 2. Human high/low hormones groups distribution \| \| \| \| \| \| \| --- \| --- \| --- \| --- \| --- \| --- \| \|  \| **Men** \| **EF** \| **LF** \| **ML** \| **OCA** \| \| High estradiol \| 10 \| 8 \| 27 \| 18 \| 2 \| \| Low estradiol \| 22 \| 15 \| 3 \| 0 \| 21 \| \| High progesterone \| 15 \| 6 \| 19 \| 18 \| 5 \| \| Low progesterone \| 18 \| 17 \| 11 \| 0 \| 19 \| \| High P.E2 ratio \| 18 \| 8 \| 11 \| 16 \| 10 \| \| Low P.E2 ratio \| 14 \| 15 \| 19 \| 2 \| 13 \| \| Data are presented as mean ± SD. FA-FE. Generalized linear model was used. EF: early follicular, LF: late follicular, ML: mid-luteal, OCA: oral contraceptive users. \| \| \| \| \| \|   Data are presented as mean ± SD or number of subjects and percentage, n (%). STAI-T: trait section from the Spanish Version of the State-Trait Anxiety Inventory-Trait version (range 0 to 60), US: unconditioned stimulus. Discomfort ratings ranged from 0 (no discomfort) to 9 (maximum discomfort). Generalized linear model or Chi-squared tests were used. EF: early follicular, LF: late follicular, ML: mid-luteal, OCA: oral contraceptive users. | | | | | | |

| Supplementary Table 3. Animals´ characteristics | | | | | | |
| --- | --- | --- | --- | --- | --- | --- |
|  | **Males** | **Proestrus** | **Estrus** | **Metestrus** | **Diestrus** | ***p-value*** |
| Age, in months | 3.4 ± 0.5 | 2.9 ± 0.4 | 3.2 ± 0.7 | 3.3 ± 0.7 | 3.3 ± 0.7 | 0.218 |
| Weight, in grams | 28.7 ± 3.2 | 19.9 ± 1.3 | 20.9 ± 3.2 | 21.5 ± 1.9 | 20.9 ± 2.5 | 0.000 |
| Days of cycle monitoring | _ | 12.1 ± 4.2 | 7.7 ± 5.3 | 12.0 ± 10.3 | 11.7 ± 8.9 | 0.297 |
| Amount of pre-extinction plasma | 71.9 ± 28.1 | 68.2 ± 9.8 | 60.7 ± 21.5 | 63.8 ± 21.3 | 58.2 ± 13.9 | 0.159 |
| Data are presented as mean ± SD. FA-FE. Generalized linear model was used. EF: early follicular, LF: late follicular, ML: mid-luteal, OCA: oral contraceptive users. | | | | | | |

| Supplementary Table 4. Animal high/low hormones groups distribution | | | | | |
| --- | --- | --- | --- | --- | --- |
|  | **Males** | **Proestrus** | **Estrus** | **Metestrus** | **Diestrus** |
| High estradiol | 6 | 10 | 8 | 5 | 15 |
| Low estradiol | 26 | 1 | 7 | 3 | 7 |
| High progesterone | 10 | 0 | 12 | 7 | 15 |
| Low progesterone | 22 | 11 | 3 | 1 | 7 |
| High P.E2 ratio | 19 | 0 | 8 | 6 | 11 |
| Low P.E2 ratio | 13 | 11 | 7 | 2 | 11 |
| Data are presented as mean ± SD. FA-FE. Generalized linear model was used. EF: early follicular, LF: late follicular, ML: mid-luteal, OCA: oral contraceptive users. | | | | | |
